# Extracellular protein components of amyloid plaques and their roles in Alzheimer’s disease pathology

**DOI:** 10.1186/s13024-021-00465-0

**Published:** 2021-08-28

**Authors:** M. Mahafuzur Rahman, Christofer Lendel

**Affiliations:** Department of Chemistry, KTH Royal Institute of Technology, SE-100 44 Stockholm, Sweden

**Keywords:** Alzheimer’s disease, Senile plaque, Amyloid-β, Protein interaction network, Amyloid corona

## Abstract

Alzheimer’s disease (AD) is pathologically defined by the presence of fibrillar amyloid β (Aβ) peptide in extracellular senile plaques and tau filaments in intracellular neurofibrillary tangles. Extensive research has focused on understanding the assembly mechanisms and neurotoxic effects of Aβ during the last decades but still we only have a brief understanding of the disease associated biological processes. This review highlights the many other constituents that, beside Aβ, are accumulated in the plaques, with the focus on extracellular proteins. All living organisms rely on a delicate network of protein functionality. Deposition of significant amounts of certain proteins in insoluble inclusions will unquestionably lead to disturbances in the network, which may contribute to AD and copathology. This paper provide a comprehensive overview of extracellular proteins that have been shown to interact with Aβ and a discussion of their potential roles in AD pathology. Methods that can expand the knowledge about *how* the proteins are incorporated in plaques are described. Top-down methods to analyze post-mortem tissue and bottom-up approaches with the potential to provide molecular insights on the organization of plaque-like particles are compared. Finally, a network analysis of Aβ-interacting partners with enriched functional and structural key words is presented.

## Background

**Fig. 1:**
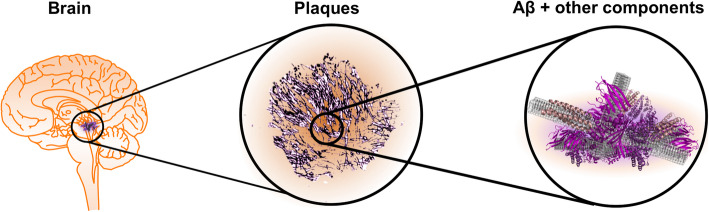
Extracellular senile plaques are pathological hallmarks of AD brains. The plaques are proteinaceous deposits with Aβ as main constituent but also containing a range of other components

Alzheimer’s disease (AD) is the most prevalent cause of dementia today, affecting close to 50 million people worldwide [1]. Considering the fact that we still lack curative treatment, AD is likely to become a serious burden on our future healthcare systems, especially with the increased life span we have experienced during the last century. The clinical symptoms include progressive memory loss, language disturbance, and mood-behavioral changes, but they are not by themselves enough to define the disease. Instead the definition relies on pathological protein inclusions, extracellular senile plaques (Fig. 1) and intracellular neurofibrillary tangles, in the patient’s brain. Hence, for long time the diagnosis could only be decided post-mortem. More recently, biomarkers in cerebrospinal fluid (CSF; and potentially also blood serum) [2, 3] and positron emission tomography (PET) imaging techniques [4] have been shown to correlate well with the protein deposition pathology. The majority of AD patients also display co-pathologies, that are not necessarily identified by biomarkers or imaging [5, 6]. It has also been shown that similar protein inclusions are often found in people without any symptoms of dementia [7], hence the disease is believed to have an extended preclinical phase. Along with pathological hallmarks of plaques and tangles, the AD pathology may be associated with cerebral amyloid angiopathy (CAA), synaptic failure, oxidative damage, neuroinflammation and mitochondrial dysfunction [8].

The connection between dementia and the pathological protein inclusions that signify AD was first reported by Alois Alzheimer in 1906 [9] and the senile plaques were defined as amyloid by the observation of Congo red binding [10]. However, it would take almost 80 years from the first report by Alzheimer before the core components of the amyloid deposits were identified; Glenner and Wong identified amyloid β (Aβ) in vascular amyloid in 1984 [11] and Masters *et al.* in plaques the year after [12]. Tau was found to be the main components of neurofibrillary tangles in 1985 by Brion *et al.* [13]. The observation of a link between mutations in the gene coding for the Aβ precursor protein (APP) and early onset familial forms of AD [14–16] suggested Aβ to be the causative agent for the disease and the ‘amyloid cascade hypothesis’ was founded. This hypothesis states that the pathology is initiated by aggregation of Aβ due to mutations making the peptide more aggregation prone or, in the expanded version, also covering sporadic AD due to imbalance in the Aβ production and degradation.

Based on the pathological and genetic findings, research around Aβ turned into a very popular field with thousands of papers published every year. Among the more important progress made within the biochemistry of AD are the determination of high-resolution structures of Aβ amyloid [17–20], the proposal of a critical role for pre-fibrillar structures (oligomers or protofibrils) [21, 22] and the dissection of the microscopic mechanisms of the amyloid formation process [23, 24]. The roles and interplay between various cell types in the central nervous system has also become evident [25]. With all this knowledge one would expect that we would also have effective therapy available, but that is not the case. The biological processes by which Aβ accumulation causes neurodegeneration are far from understood and widely debated. This calls for generation of even more knowledge and exploration of new hypotheses. With this article we want to shift the focus from the Aβ core component of the plaques to the many other proteins that co-aggregate with Aβ amyloid. We first describe methods to investigate the composition of plaques from top-down and bottom-up perspectives. Then we present an overview of extracellular proteins that have been found to interact with aggregated A*β in vitro* and highlight potential connections to AD pathology reported in the literature.

## Top-down approaches to identify protein components of plaques

The identification of Aβ in senile plaques from post-mortem tissue was indeed a breakthrough in the molecular description of AD. However, Aβ was not the first protein to be associated with plaques. Immunoglobulins and complement proteins had already been detected [26, 27]. In 1994, at least 35 protein components had been found to be associated with senile plaques [28], including apolipoprotein E (apoE), clusterin, vitronectin, coagulation factors, heat shock proteins, proteases and protease inhibitors. Hence, apoE was indeed identified as a senile plaque component [29] before it was found to be an important genetic risk factor of AD [30].

**Fig. 2:**
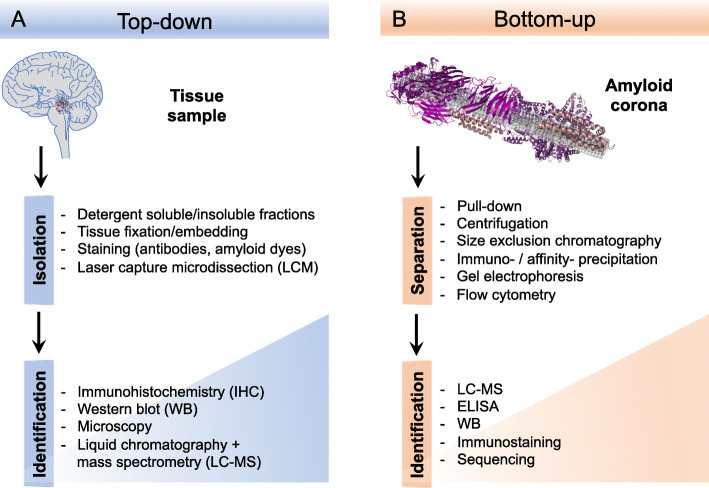
Approaches to explore the composition and organization of senile plaques. (**A**) Top-down methods starts from plaque tissue samples and analyze the plaque structure by *e.g.* microscopy or mass-spectrometry proteomics. (**B**) Bottom-up methods make use of *in vitro* models in order to study composition, protein structure and interactions from a molecular perspective. The amyloid corona refers to the layer of proteins from a biological fluid that is sequestered by the amyloid fibrils

For the early identification studies, analysis of samples from dedicated purification protocols or immunohistochemical investigation of tissues were used (Fig. 2A). Although these can give clear and reliable results, they are limited to availability and the quality of antibodies and one need to know what to look for in order to find it. The emergence of effective proteomics techniques and in particular mass spectrometry (MS), allowed broader, unbiased investigations of the plaque components. On the other hand, the sample preparation methods become increasingly important and the question what is “in the plaque” and what originates from the surrounding tissue stands out as very critical. Chemical purification methods using harsh conditions may remove too much of the plaque associated components while common methods such as laser capture microdissection (LCM) would include everything within the specified area. The problems can, to some extent, be overcome by appropriate controls, *e.g.* comparison with LCM of tissue just outside the plaques [31, 32] or with samples from different purification protocols, but still the methods report on spatial proximity rather than structural or molecular connections.

Increased sensitivity of MS instrument has also lead to an increasing number of identified proteins. The pioneering study by Liao *et al.* in 2004 reported 488 proteins found in plaques [31] while Xiong and co-workers in 2019 and Bai and co-workers in 2020 were able to detect more than 4000 and 14,000 proteins, respectively [32, 33]. Notably, a study published 2 years after Liao *et al.*, and employing a different sample preparation protocol, only found Aβ in the plaque cores [34]. Hence, the employed methodology can have substantial effect on which plaque components that are identified.

Investigations of post-mortem tissues are extremely important since they are authentic samples. There are endless opportunities to compare different tissues, different classes of plaques, different individuals etc. This is important since the molecular assembly of all plaque types is not the same [5, 35]. The *neuritic* or *dense core plaques* are formed around a core of fibrillar Aβ structure and have neighboring reactive astrocytes and activated microglial cells. *Diffuse plaques* are poorly marginated assemblies of filamentous Aβ, which is weakly stained by amyloid-specific dyes, and do not have associated toxicities as the neuritic plaque. The diffuse plaques often lack of neuritic elements, nevertheless, *diffuse neuritic plaques* can be observed in advanced AD. Yet another class are the cerebrovascular Aβ deposits, in the course of CAA. There are, however, limitations in the possibilities to acquire a detailed molecular understanding of the plaque structures. For examples, which components are directly sequestered by Aβ amyloid fibrils, which components may be more loosely bound or bound to some of the already sequestered proteins and which components just happened to be localized in vicinity of the plaques? Crosslinking protocols could potentially resolve some of these question [36, 37]. Such studies have been carried out to identify binders of tau and APP in murine models [38, 39] but not yet to explore the architecture of plaques.

Microscopy offers many opportunities to analyze the presence of different components and their structural properties with spatial resolution. Combining specific antibodies and various amyloid probes with conformation-dependent optical properties allows for imaging of heterogeneities within single plaques (see *e.g.* Ref. [40] for illustration). Confocal imaging allows for 3D reconstruction of the plaque structures. However, these studies are, as all microscopy techniques, limited by the diffraction laws that set a size limit in the micrometer range. Even though new super-resolution techniques have pushed this limit towards the nanometer range, it is still not possible to derive molecular information about the deposits. Cryo-electron microscopy has become an increasingly important method for the structural characterization of amyloid fibrils [41] allowing resolution below nanometer range. Moreover, cryo-electron tomography has recently provided impressive 3D images of *in situ* deposits of fibrillar proteins that also include organelle structures such as endoplasmic reticulum, ribosomes and proteasomes [42, 43]. Protein-specific information, *e.g.* for investigating co-localization, can also be achieved in electron microscopy through immunolabeling with colloidal gold. Hence, imaging of senile plaques with molecular resolution may soon give us the first glimpses of the arrangement of the different molecular components.

Proper knowledge of the structural and functional connections within the plaques is likely as important (or even more) for the understanding of the pathology as a detailed understanding of the Aβ amyloid formation process. Any pathological process triggered by Aβ (as monomer, oligomer or amyloid fibrils) must proceed via interactions with other biomolecules. Hence, it is not enough to know which proteins are found in the plaques (or close to them) we must also decipher how they are organized and what functional consequences the deposition has for each protein, process and biological pathway. Therefore, biochemical model systems that can capture these effects are, and will continue to be, important for the progress of AD research.

## Bottom-up approaches to identify interaction partners of amyloid

There are many methods that enable *in vitro* studies of interactions between biomolecules and with the opportunity to derive structural as well as functional parameters. One of the challenges when approaching systems, such as senile plaques, is the complexity in terms of the number of different components. There is always a balance between the simplicity of an *in vitro* model and its ability to capture biologically relevant features. If we accept that Aβ fibril formation is a central process in the pathology, that should also be the starting point for building *in vitro* models. Studies of Aβ fibrillation have been a popular field for quite some time. The progress in these studies has provided in-depth understanding of how Aβ, and proteins in general, assemble into amyloid fibrils. Although the experiments are typically carried out in pure systems with the addition of only a few additional components, the mechanism rapidly become very complex. Fibrillation of Aβ in *in vivo*–like environments, *e.g.* CSF [44], has demonstrated that the kinetics is affected, indicating that the amyloid interacts with other components. However, it remains to be described which roles the different components play and potential synergistic effects. We therefore need ways to map out which components are involved (Fig. 2B).

In a pioneering study from 2012, Olzscha *et al.* studied intracellular protein aggregation in a cell model and found a correlation between the toxicity of artificial β-sheet proteins and their ability to sequester cellular proteins [45]. Experiments with Aβ in the same model system confirmed that also these aggregates attracted a range of protein binding partners. Many of the sequestered proteins were hubs in functional cellular networks indicating the ability of amyloids to trigger multifactorial toxic responses. The question of which proteins from a physiological environment that are sequestered to amyloid aggregates and how that affect the biological response (toxicity) of the aggregates has some similarities with a central question in another field of research: the biological effects of synthetic nanoparticles. A nanoparticle that enters a biological system will be covered with proteins, creating a “corona”, and it is the structural and functional properties of these proteins that to a large extent determine the biological effects of the nanoparticle [46, 47]. Recent studies have highlighted the fact that these features are not unique for synthetic nanoparticles. Viruses were shown to attract protein coronae when introduced into various biological fluids, such as human plasma or human bronchoalveolar lavage fluid [48]. Interestingly, the viruses also seemed to accelerate the aggregation of Aβ into amyloid, which suggest a potential role of heterogenous nucleation in amyloid diseases [49]. In another study, we demonstrated that pre-fibrillar Aβ aggregates (protofibrils) attract a range of different proteins when exposed to human serum or CSF [50]. In a follow-up study, proteins binding to Aβ amyloid fibrils were investigated and fibrils were found to sequester more proteins than the protofibrils and with distinct functional characteristics [51]. Other studies have confirmed the formation of protein coronae around Aβ fibrils and for amyloid fibrils of IAPP, α-synuclein, a C-terminal fragment of α_1_-antitrypsin, the FAS4 domain from human cornea and the human hormone glucagon [52–54].

The amyloid corona concept offers a new and interesting perspective on the assembly of plaque-like multiprotein aggregates and could potentially be the starting point for studying plaque formation with molecular resolution. However, the methods to identify, analyze and quantify the components need to be developed and advantages and drawbacks of each approach evaluated. Common separation methods are filtration [53, 54], centrifugation [52] and pull-down assays using magnetic beads [50, 51]. New applications of old techniques could also offer opportunities in this field. Madasamy *et al.* demonstrated that flow-cytometry could be used to isolate “plaque particles” formed around Aβ, α-synuclein, tau or cholesterol added to serum samples [55]. In addition to the added “seeds”, almost 200 serum proteins were identified in the particles. We recently developed this approach further and showed that it may have advantages over pull-down methods or at least provide complementary perspectives on the composition of Aβ amyloid coronae [56]. A central finding in that study is that not all protein bind directly to Aβ. This illustrate that the assembly is more complex than just sequestration of the proteins by the amyloid structure. Moreover, the recent study from Nandakumar *et al.* [53] shows that the protein corona around Aβ amyloid can mask the antibody binding epitopes on Aβ, which highlights the need to characterize the structural properties of multiprotein amyloid aggregates to obtain a better understanding of the potential for amyloid-targeted immunotherapy.

Taken together, it is clear that there is an emerging interest to explore the composition and organization of amyloid protein coronae, which opens opportunities to acquire new knowledge about the multicomponent structures of senile plaques and new insights about AD pathology.

## Survey of extracellular proteins that associate with Aβ

As already stated, thousands of proteins have been identified in post-mortem plaque tissue. A comprehensive review of these proteins is not feasible. Instead we chose to apply a bottom-up perspective and investigate the set of proteins found by recent *in vitro* studies as Aβ-interacting proteins in human biofluids, including plasma, serum, and CSF. These studies have reported more than a hundred different proteins with the ability to interact with different conformations of Aβ. The majority of these proteins are also known to be present in senile plaques. As the survey is limited to proteins from biofluids, cell-surface receptors and intracellular proteins are naturally excluded unless they are also secreted to the fluid. Nevertheless, a brief discussion of these proteins is included in a separate section. The plaques also contain many non-protein components, including proteoglycans, nucleic acids, lipids and metal ions. For more details about these classes of molecules we refer to recent reviews: [57, 58].

In this section, we will discuss the implicatiyuulators of Aβ fibrillogenesis. The proteins are categorized and presented according to their main biological functions. We have listed the proteins that are found in the literature to interact with Aβ in *at least two separate studies* in Table 1 with notes on the isoform and conformation of Aβ, methodology used, and binding affinity. Furthermore, confirmed existence in senile plaque as well as the reported potential as AD biomarker are indicated.

**Table 1:** Biochemical and biophysical evidence of interactions of Aβ with other proteins, and their presence in senile plaque and potential for AD biomarker

Protein name	Bound to Aβ *in vitro*		Found in post-mortem AD plaque and CAA		Candidate biomarker for AD
	Aβ monomer, oligomers, and protofibrils	Aβ fibrils			
			Reported by	Brain region; and plaque types/CAA	
**Immunoglobulins**					
Ig alpha-1 chain C region	[50]^a^	[52, 56]^b,d^	[32, 33, 59, 60]^f,j†,g,h^	HC^f^, FCtx^j†^, HC^g^, CCtx and Cve^h^	[61]:↑P [62]:↑C
Ig gamma-1 chain C region	[50]^a^	[51, 52, 56]^e,b,d^	[26, 32, 33, 59]^i,f,j†,g^	F-tCtx^i^, HC^f^, FCtx^j†^, HC^g^	[61]:↓P
Ig gamma-2 chain C region	[50]^a^	[52, 56]^b,d^	[26, 32, 33, 59]^i,f,j†,g^	F-tCtx^i^, HC^f^, FCtx^j†^, HC^g^	
Ig gamma-3 chain C region	[50]^a^	[52, 56]^b,d^	[26, 32, 33, 59]^i,f,j†,g^	F-tCtx^i^, HC^f^, FCtx^j†^, HC^g^	
Ig kappa chain C region	[50]^a^	[51, 52, 56]^e,b,d^	[26, 32, 33, 59, 60]^i,f,j†,g,h^	F-tCtx^i^, HC^f^, FCtx^j†^, HC^g^, CCtx and Cve^h^	[61]:↑P
Ig mu chain C region	[50]^a^	[51, 52, 56]^e,b,d^	[32, 33]^f,j†^	HC^f^, FCtx^j†^	[61]:↓P
Immunoglobulin heavy constant alpha 2		[51, 56]^e,d^			
Immunoglobulin heavy variable 3-7		[51, 53]^e,j^			
**Complement system**					
Alpha-1-antichymotrypsin	[50, 63–65]^a,k,l,n^	[56]^d^	[28, 31, 32, 59, 60, 66, 67]^q,r,f,g,h,s,t^	FCtx and TCtx^r^, HC^f,g^, CCtx and Cve^h^, PreFCtx^s^	[68]:↑P [69]:↑C
C4b-binding protein alpha chain		[52, 53, 56]^b,j,d^	[28, 32, 33]^q,g,j†^	HC^f^, FCtx^j†^	
Complement C1q subcomponent subunit A/subunit B/subunit C	[50, 70]^a,u^	[51, 56, 71, 72]^e,d,v,w^	[26, 28, 31–33, 59, 60, 66, 73]^i,q,r,f,g,j†,h,s,x^	F-tCtx^i^, FCtx and TCtx^r^, HC^f^, FCtx^j†^, HC^g^, CCtx and Cve^h^, PreFCtx^s^, TLb^x^; CP^x^	[74]:↓C (subunit B/C)
Complement C1r subcomponent		[51–53, 56]^e,b,j,d^	[32, 33]^f,j†^	HC^f^, FCtx^j†^	
Complement C1s subcomponent		[51–53, 56]^e,b,j,d^	[32, 33]^f,j†^	HC^f^, FCtx^j†^	[74]:↓C
Complement C3	[50]^a^ 0.3 μM (Aβ42PF)^a^	[51–53, 56, 72, 75]^e,b,j,d,w,y^	[26, 28, 31–33, 59, 60, 66, 73]^i,q,r,f,j†,g,h,s,x^	F-tCtx^i^, FCtx and TCtx^r^, HC^f^, FCtx^j†^, HC^g^, CCtx and Cve^h^, PreFCtx^s^, TLb^x^; CP and DP^x^	[76, 77]:↑P/S, [78]:↑C
Complement C4-A/C4-B	[50]^a^	[51–53, 56, 72]^e,b,j,d,w^	[26, 31, 32, 59, 60, 66, 73] ^i,r,f,g,h,s,x^	F-tCtx^i^, FCtx and TCtx^r^, HC^f,g^, CCtx and Cve^h^, PreFCtx^s^, TLb^x^; CP and DP^x^	[61, 76]:↑↓S, [79]:↑C
Complement C5		[53, 56, 72]^j,d,w^	[32, 33]^f,j†^	HC^f^, FCtx^j†^	
Complement component C7		[51, 53, 56]^e,j,d^	[32, 33]^f,j†^	HC^f^, FCtx^j†^	
Complement component C8 gamma chain		[53, 56]^j,d^	[32, 33, 60]^f,j†,h^	HC^f^, FCtx^j†^, CCtx and Cve^h^	
Complement component C9		[51, 53, 56]^e,j,d^	[32, 33]^f,j†^	HC^f^, FCtx^j†^	
Complement factor H		[51–53, 56]^e,b,j,d^	[32, 33, 59]^f,j†,g^	HC^f^, FCtx^j†^, HC^g^	[61, 76]:↑P/S
Complement factor H-related protein 5	[50]^a^	[51, 56]^e,d^			
Inter-alpha-trypsin inhibitor heavy chain H4	[50]^a^	[51, 53, 56]^e,j,d^	[32, 33]^f,j†^	HC^f^, FCtx^j†^	[61]:↓P, [80]:↑C
Monocyte differentiation antigen CD14	[50]^a^	[51, 56]^e,d^	[32, 33]^f,j†^	HC^f^, FCtx^j†^	
Plasma protease C1 inhibitor		[51, 56]^e,d^	[32, 33, 60]^f,j†,h^	HC^f^, FCtx^j†^, CCtx and Cve^h^	
**Lipid metabolism/transport**					
Apolipoprotein A-I	[50, 81–84]^a,z,a†,b†,d†^ 3 μM (Aβ42PF)^a^, 6 nM (Aβ40M)^d†^	[51–53, 56, 85]^e,b,j,d,e†^	[32, 33, 59, 60, 66, 86]^f,j†,g,h,s,f†^	HC^f^, FCtx^j†^, HC^g,^, CCtx and Cve^h^, PreFCtx^s^, HC^f†^	[87–89]:↓ S/P, [90]:↑C
Apolipoprotein A-II	[50, 82]^a,a†^	[51–53, 56, 85]^e,b,j,d,e†^	[32, 33]^f,j†^	HC^f^, FCtx^j†^	
Apolipoprotein A-IV	[50, 83]^a,b†^	[51–53, 56]^e,b,j,d^	[32, 33]^f,j†^	HC^f^, FCtx^j†^	[88]:↑P, [80]: ↑C
Apolipoprotein B-100	[50]^a^	[52]^b^	[32, 33, 60]^f,j†,h^	HC^f^, FCtx^j†^, CCtx and Cve^h^	[76, 89]:↑↓S
Apolipoprotein C-I		[51, 52, 56]^e,b,d^			[91]:↓C
Apolipoprotein C-II	[92]^g†^	[51, 52]^e,b^	[32, 33]^f,j†^	HC^f^, FCtx^j†^	
Apolipoprotein C-III		[51–53, 56]^e,b,j,d^	[32, 33, 59]^f,j†,g^	HC^f^, FCtx^j†^, HC^g^	
Apolipoprotein C-IV	[50]^a^	[51, 56]^e,d^			
Apolipoprotein D	[50]^a^	[51, 52, 56]^e,b,d^	[32, 33, 59, 60, 66, 93, 94]^f, j†,g,h,s,h†,i†^	HC^f^, FCtx^j†^, HC^g^, CCtx and Cve^h^, PreFCtx^s^, CCtx and HC^h†,i†^; DP, around CP, and CAA^h†,i†^	[95, 96]:↓C
Apolipoprotein E	[50, 63, 82–84]^a,k,a†,b†,d†^ 3 nM (Aβ42PF)^a^, 19 nM (Aβ40M)^d†^	[51–53, 56, 85]^e,b,j,d,e†^	[28, 31–33, 59, 60, 66, 94] ^q,r,f,j†,g,h,s,i†^	FCtx and TCtx^r^, HC^f^, FCtx^j†^, HC^g^, CCtx and Cve^h^, PreFCtx^s^, CCtx and HC^i†^; CP, DP, and CAA^i†^	[76]:↑S, [78, 97, 98]:↑↓C
Apolipoprotein L1		[51, 53, 56]^e,j,d^	[32, 33]^f,j†^	HC^f^, FCtx^j†^	
Beta-2-glycoprotein 1		[51, 56]^e,d^	[32, 33]^f,j†^	HC^f^, FCtx^j†^	
Clusterin (a.k.a, ApoJ)	[50, 81–83, 99, 100]^a,z,a†,b†,k†,l†^ 4.8 nM (Aβ40M)^k†^, 2 nM (AβM)^l†^	[51–53, 56, 85]^e,b,j,d,e†^	[28, 31–33, 59, 60, 66, 73, 101]^q,r,f,j†,g,h,s,x,n†^	FCtx and TCtx^r^, HC^f^, FCtx^j†^, HC^g^, CCtx and Cve^h^, PreFCtx^s^, TLb^x^, EntCtx^n†^;CP^x,n†^	[102]:↓P, [78]:↑C
Phospholipid transfer protein	[50]^a^	[51, 56]^e,d^	[32, 33]^f,j†^	HC^f^, FCtx^j†^	
Prostaglandin-H2 D-isomerase	[50]^a^	[51, 56]^e,d^	[32, 59, 60, 66]^f,g,h,s^	HC^f,g^, CCtx and Cve^h^, PreFCtx^s^	[62, 103]:↑↓C
Serum amyloid A-4 protein	[50]^a^	[51, 53, 56]^e,j,d^	[32, 33]^f,j†^	HC^f^, FCtx^j†^	
**Blood coagulation/hemostasis**					
Adipocyte enhancer-binding protein 1		[51, 56]^e,d^			
Alpha-1-antitrypsin	[50]^a^	[51, 52, 56]^e,b,d^	[28, 31–33, 59, 60, 66]^q,r,f,j†,g,h,s^	FCtx and TCtx^r^, HC^f^, FCtx^j†^, HC^g^, CCtx and Cve^h^, PreFCtx^s^	[89, 102, 104]:↑↓ P/S, [80, 96]:↑↓C
Alpha-2-macroglobulin	[84, 105, 106]^d†,r†,s†^ 0.34 μM (Aβ40M)^d†^, 0.35 μM (Aβ40M)^r†^, 38 μM (Aβ42M)^s†^	[53, 56]^j,d^	[28, 31–33, 59, 60, 66]^q,r,f,j†,g,h,s^	FCtx and TCtx^r^, HC^f^, FCtx^j†^, HC^g^, CCtx and Cve^h^, PreFCtx^s^	[61, 76, 107]:↑ P/S
Angiotensinogen	[50]^a^	[51, 56]^e,d^	[32, 33, 59]^f,j†,g^	HC^f^, FCtx^j†^, HC^g^	
Antithrombin-III	[50]^a^ 0.6 μM (Aβ42PF)^a^	[51, 53, 56]^e,j,d^	[32, 33, 59, 108]^f,j†,g,t†^	HC^f^, FCtx^j†^, HC^g^, CCtx^t†^; CP^t†^	[69, 80]:↑C
Beta-1,4-glucuronyltransferase1		[51, 56]^e,d^	[32, 59]^f,g^	HC^f,g^	
Carboxypeptidase B2		[53, 56]^j,d^	[33]^j†^	FCtx^j†^	
Coagulation factor V	[50]^a^	[51, 56]^e,d^	[33]^j†^	FCtx^j†^	
Coagulation factor X		[51, 52, 109]^e,b,u†^	[33]^j†^	FCtx^j†^	
Coagulation factor XII	[110]^v†^	[53, 56, 111]^j,d,w†^	[32, 33, 66]^f,j†,s^	HC^f^, FCtx^j†^, PreFCtx^s^	
Fibrinogen alpha−, beta−, and gamma−chain	[50, 112]^a,x†^ 26 nM (Aβ42M)^x†^	[51, 52, 56]^e,b,d^	[31–33, 59, 60, 66, 113]^r,f,j†,g,h,s,y†^	FCtx and TCtx^r^, HC^f^, FCtx^j†^, HC^g^, CCtx and Cve^h^, PreFCtx^s^, FCtx^y†^; CAA^y†^	[77, 88, 89]:↑↓ P/S, [98]:↑C
Fibronectin	[50]^a^	[51–53, 56, 109]^e,b,j,d, u†^	[32, 33, 59, 66]^j†,f,g,s^	HC^f^, FCtx^j†^, HC^g^, PreFCtx^s^	[89]:↑S
Growth arrest-specific protein 6		[51, 56]^e,d^	[33]^j†^	FCtx^j†^	[114]:↑C
Heparin cofactor 2	[50]^a^	[51, 56]^e,d^	[32, 33]^f,j†^	HC^f^, FCtx^j†^	
Histidine-rich glycoprotein	[50]^a^	[52, 56]^b,d^	[32, 33, 59]^j†,f,g^	FCtx^j†^, HC^f,g^	[76]:↑S
Hyaluronan-binding protein 2		[52, 56]^b,d^	[31–33]^r,f,j†^	FCtx and TCtx^r^, HC^f^, FCtx^j†^	
Kininogen-1		[51, 53, 56]^e,j,d^	[32, 33]^f,j†^	HC^f^, FCtx^j†^	
Plasminogen		[51, 53, 109]^e,j,u†^	[32, 33]^f,j†^	HC^f^, FCtx^j†^	[78]:↑C [88]:↓P
Prothrombin	[50]^a^	[51–53, 56]^e,b,j,d^	[32, 33]^f,j†^	HC^f^, FCtx^j†^	
Vitamin K-dependent protein S		[51–53, 56]^e,b,j,d^	[32, 33]^f,j†^	HC^f^, FCtx^j†^	
**Metabolism**					
Alpha-enolase	[50]^a^	[51, 56]^e,d^	[32, 33, 59, 60, 66, 115]^f,j†,g,h,s,z†^	HC^f^, FCtx^j†^, HC^g^, CCtx and Cve^h^, PreFCtx^s^	
Glyceraldehyde-3-phosphate dehydrogenase	[50, 63]^a,k^	[51, 56, 116]^e,d,a‡^	[31, 32, 59, 60, 66]^r,f,g,h,s^	FCtx and TCtx^r^, HC^f,g^, CCtx and Cve^h^, PreFCtx^s^	
Phosphoglycerate kinase 1	[50]^a^	[56]^d^	[31–33, 59, 60, 66]^r,f,j†,g,h,s^	FCtx and TCtx^r^, HC^f^, FCtx^j†^, HC^g^, CCtx and Cve^h^, PreFCtx^s^	
Procollagen C-endopeptidase enhancer 1	[50]^a^	[51, 56]^e,d^			
**Molecular transport**					
Haptoglobin	[50]^a^	[53, 56]^j,d^	[32, 33, 59, 60, 66]^f,j†,g,h,s^	HC^f^, FCtx^j†^, HC^g^, CCtx and Cve^h^, PreFCtx^s^	[76]:↑S
Hemoglobin subunit alpha/subunit beta	[50]^a^	[51, 56]^e,d^	[31–33, 59, 60, 66]^r,f,j†,g,h,s^	FCtx and TCtx^r^, HC^f^, FCtx^j†^, HC^g^, CCtx and Cve^h^, PreFCtx^s^	[76]:↑S
Hemopexin		[53, 56]^j,d^	[32, 33, 59]^f,j†,g^	HC^f^, FCtx^j†^, HC^g^	[104]:↑P
Inter-alpha-trypsin inhibitor heavy chain H2	[50]^a^	[53, 56]^j,d^	[32, 33]^f,j†^	HC^f^, FCtx^j†^	
Serotransferrin	[50]^a^	[53, 56]^j,d^	[31–33, 59, 66]^r,f,j†,g,s^	FCtx and TCtx^r^, HC^f^, FCtx^j†^, HC^g^, PreFCtx^s^	
Serum albumin	[50, 83, 117–120]^a,b†, b‡,c‡,d‡,e‡^ 1–100 nM (Aβ40O)^b‡^, 1.7 μM (Aβ42M)^c‡^, 5 μM (Aβ40M)^d‡^, ~0.1–1 mM (Aβ40M)^e‡^	[51–53, 56, 109]^e,b,j,d,u†^	[31–33]^r,f,j†^	FCtx and TCtx^r^, HC^f^, FCtx^j†^	[61]:↑P, [62, 98]:↑↓C
Transthyretin	[81, 121]^z,f‡^ ~28 nM (Aβ40M/O)^f‡^	[51, 52, 56, 121] [122]^e,b,d,f‡,h‡^ ~28 nM (Aβ42F)^f‡^	[32, 33, 66]^f,j†,s^	HC^f^, FCtx^j†^, PreFCtx^s^	[76]:↑S, [78]:↑C
Vitamin D-binding protein		[51–53, 56]^e,b,j,d^	[32, 33, 59]^f,j†,g^	HC^f^, FCtx^j†^, HC^g^	[102]:↑P, [78]:↑C
**Neural proteins**					
Amyloid-like protein 1		[51, 52, 56]^e,b,d^	[32, 33, 59]^f,j†,g^	HC^f^, FCtx^j†^, HC^g^	[62, 123]:↑↓C
Brevican core protein		[51, 109]^e,u†^	[32, 33, 59, 60, 66]^f,j†,g,h,s^	HC^f^, FCtx^j†^, HC^g^, CCtx and Cve^h^, PreFCtx^s^	
Neural cell adhesion molecule 1		[51, 56]^e,d^	[31–33, 59, 60, 66]^r,f,j†,g,h,s^	FCtx and TCtx^r^, HC^f^, FCtx^j†^, HC^g^, CCtx and Cve^h^, PreFCtx^s^	[103]:↑C
Neurocan core protein		[51, 109]^e,u†^ 11.7 nM (Aβ42F)^e^	[32, 33, 59, 60, 66]^f,j†,g,h,s^	HC^f^, FCtx^j†^, HC^g^, CCtx and Cve^h^, PreFCtx^s^	
Neurosecretory protein VGF		[51, 56]^e,d^	[32, 33, 59, 66]^f,j†,g,s^	HC^f^, FCtx^j†^, HC^g^, PreFCtx^s^	[79, 90, 124, 125]:↓C
ProSAAS		[51, 52, 56]^e,b,d^	[32, 33, 59, 60, 66]^f,j†,g,h,s^	HC^f^, FCtx^j†^, HC^g^, CCtx and Cve^h^, PreFCtx^s^	[62, 78]:↑↓C
**Cell adhesion, extracellular matrix, and proteoglycans**					
Agrin		[51, 109, 126]^e,u†,i‡^ 3.5 nM (Aβ42F)^e^	[32, 33, 59, 60, 66, 126]^f,j†,g,h,s,j‡^	HC^f^, FCtx^j†^, HC^g^, CCtx and Cve^h^, PreFCtx^s^, FCtx^j‡^; CP, DP, and CCA ^j‡^	[127]^k‡^
Basement membrane-specific heparan sulfate proteoglycan core protein		[51, 56]^e,d^	[32, 33, 59, 60, 66]^f,j†,g,h,s^	HC^f^, FCtx^j†^, HC^g^, CCtx and Cve^h^, PreFCtx^s^	
Cartilage acidic protein 1	[50]^a^	[51, 56]^e,d^	[32, 33, 59, 60]^f,j†,g,h^	HC^f^, FCtx^j†^, HC^g^, CCtx and Cve^h^	
Collagen alpha-1(XVIII) chain	[50]^a^	[51, 56]^e,d^	[32, 33, 59, 66]^f,j†,g,s^	HC^f^, FCtx^j†^, HC^g^, PreFCtx^s^	
Decorin	[50, 128]^a,l‡^	[51, 56, 109]^e,d,u†^	[32, 33, 129]^f,j†,n‡^	HC^f^, FCtx^j†^, HC^n‡^; CP^n‡^	
Desmoplakin	[50]^a^	[51, 56]^e,d^	[32, 33, 59, 66]^f,j†,g,s^	HC^f^, FCtx^j†^, HC^g^, PreFCtx^s^	[61]:↑P
EGF-containing fibulin-like extracellular matrix protein 1	[50]^a^	[51, 52]^e,b^	[32]^f^	HC^f^	[74]:↓C
Extracellular matrix protein-1		[53, 109]^j,u†^	[32]^f^	HC^f^	
Extracellular matrix protein-2	[50]^a^	[51, 56]^e,d^	[32]^f^	HC^f^	
Fibulin-1	[50, 83]^a,b†^	[51–53, 56]^e,b,j,d^	[32]^f^	HC^f^	[89]:↑S
Galectin-3-binding protein	[50]^a^	[51, 56]^e,d^	[32, 33, 60, 66]^f,j†,h,s^	HC^f^, FCtx^j†^, CCtx and Cve^h^, PreFCtx^s^	
Glypican-1		[51, 109, 130]^e,u†q‡^	[32, 33, 59]^f,j†,g^	HC^f^, FCtx^j†^, HC^g^	
Microfibril-associated glycoprotein 4	[50]^a^	[56]^d^	[33, 66]^j†,s^	FCtx^j†^, PreFCtx^s^	
Mimecan	[50]^a^	[51, 52, 56]^e,b,d^	[33, 66]^j†,s^	FCtx^j†^, PreFCtx^s^	
Osteomodulin		[51, 56]^e,d^			
Osteopontin		[51, 56]^e,d^	[32, 33]^f, j†^	HC^f^, FCtx^j†^	[123]:↑C
Prolargin	[50]^a^	[51, 56]^e,d^	[32, 33]^f, j†^	HC^f^, FCtx^j†^	
SPARC-like protein 1		[51, 52, 56, 109]^e,b,d,u†^ 6.2 nM (Aβ42F)^e^	[32, 33, 59, 60]^f,j†,g,h^	HC^f^, FCtx^j†^, HC^g^, CCtx and Cve^h^	[98]:↑C
Vitronectin	[50, 83]^a,b†^	[51–53, 56]^e,b,j,d^	[32, 33, 59, 101, 131]^f,j†,g,n†,s‡^	HC^f^, FCtx^j†^, HC^g^, EntCtx^n†,s‡^; CP^n†,s‡^	[76]:↑S
**Other proteins**					
Actin, cytoplasmic 1	[50]^a^	[51, 56]^e,d^	[32, 59, 60, 115]^f,g,h,z†^	HC^f,g^, CCtx and Cve^h^	
Alpha-1B-glycoprotein		[53, 56]^j,d^	[32, 33]^f,j†^	HC^f^, FCtx^j†^	
Alpha-2-HS-glycoprotein		[51–53, 56]^e,b,j,d^	[32]^f^	HC^f^	[62, 79]:↓C, [88]:↓P
Beta-Ala-His dipeptidase		[51, 56]^e,d^	[32, 33]^f,j†^	HC^f^, FCtx^j†^	[90]:↓C
Cystatin-C	[50, 132, 133]^a,t‡,u‡^ 11–17 nM (Aβ40/42M)^u‡^	[51, 52, 56]^e,b,d^	[31–33, 59, 60, 66]^r,f,j†,g,h,s^	FCtx and TCtx^r^, HC^f^, FCtx^j†^, HC^g^, CCtx and Cve^h^, PreFCtx^s^	[91, 96, 97]:↑↓C
Dermcidin	[50]^a^	[51, 56]^e,d^	[31–33, 59, 66]^r,f,j†,g,s^	FCtx and TCtx^r^, HC^f^, FCtx^j†^, HC^g^, PreFCtx^s^	
Dickkopf-related protein 3		[51, 52, 56]^e,b,d^ 26.2 nM (Aβ42F)^e^	[32, 33, 59, 134]^f,j†,g,r‡^	HC^f^, FCtx^j†^, HC^g,r‡^; CP and DP^r‡^	[134]^v‡^
Gelsolin	[50, 135–137]^a,w‡,x‡,y‡^ 1.38 μM (Aβ40M)^w‡^	[51–53, 56]^e,b,j,d^	[31–33, 59, 60, 66]^r,f,j†,g,h,s^	FCtx and TCtx^r^, HC^f^, FCtx^j†^, HC^g^, CCtx and Cve^h^, PreFCtx^s^	[89]:↑S
Hepatocyte growth factor activator		[53, 56]^j,d^	[33]^j†^	FCtx^j†^	
Latent-transforming growth factor beta-binding protein 4	[50]^a^	[51, 56]^e,d^	[32]^f^	HC^f^	
Protein AMBP		[51, 53, 56]^e,j,d^	[32, 33]^f, j†^	HC^f^, FCtx^j†^	
Olfactomedin-like protein 3	[50]^a^	[51, 56]	[32, 33, 59]^f,j†,g^	HC^f^, FCtx^j†^, HC^g^	
Secreted frizzled-related protein 3		[51, 56]^e,d^	[32, 33]^f,j†^	HC^f^, FCtx^j†^	
Secretogranin-1		[51, 56]^e,d^	[32, 33, 59]^f,j†,g^	HC^f^, FCtx^j†^, HC^g^	[62, 138]:↓C
Serum amyloid P-component	[50, 83, 139]^a,b†z‡^ 6 nM (Aβ40M)^z‡^	[51, 56]^e,d^	[28, 32, 33, 59, 60, 66]^q,f,j†,g,h,s^	HC^f^, FCtx^j†^, HC^g^, CCtx and Cve^h^, PreFCtx^s^	[61]:↑P

Abbreviations used in Table:

*C* Cerebrospinal fluid, *CCtx* Cerebral cortex, *CP* Core plaque, *CVe* Cerebral vessel, *DP* Diffuse plaque, *EntCtx* Entorhinal cortex, *F* Fibrils, *FCtx* Frontal cortex, *F-tCtx* Fronto-temporal cortex, *HC* Hippocampus, *M* Monomer, *O* Oligomers, *P* Plasma, *PF* Protofibrils, *PreFCtx* Prefrontal cortex, *S* Serum, *TCtx* Temporal cortex, *TLb* Temporal lobe

Table note:

^a^Aβ42PF; pull-down from serum/CSF, LC-MS/MS, SPR. ^b^Aβ40; incubation with plasma/CSF, SDS-PAGE, LC-MS/MS. ^d^Aβ40F, Aβ42F; flow cytometry sorting from serum/CSF, LC-MS/MS. ^e^Aβ42F; pull-down from CSF, LC-MS/MS, SPR. ^f,g,h^LCM; LC-MS/MS. ^i^Immune-based detection. ^j^Aβ40; plasma protein corona, LC-MS/MS. ^k^Aβ40M; affinity isolation from rat brain, immunostaining, sequencing. ^l^Aβ42M; SDS-stable complex, WB. ^n^Aβ42M; incubation, gel electrophoresis. ^q,r^LCM; LC-MS/MS, immune-based. ^s^Detergent-insoluble plaque material; LC-MS/MS. ^t^Molecular cloning, immune-based detection. ^u^Aβ28M, Aβ38M; dot blots. ^v^Aβ42F; EM, ELISA. ^w^Aβ42F; incubation with serum, WB. ^x^Immune-based detection. ^y^Aβ42F; incubation with serum, WB. ^z^Aβ40M; SDS-stable complex, immune- and affinity-precipitation. ^a†^Aβ40M; incubation with plasma, SEC, immunostaining. ^b†^Aβ40M; affinity isolation from serum. ^d†^Aβ40M; ELISA. ^e†^Aβ; centrifugal isolation from serum, MS, WB. ^f†^Immune-based detection. ^g†^Aβ40M; pull-down from plasma, LC-MS/MS. ^h†– i†^Immune-based detection. ^j†^Tandem mass tag labeling, LC/LC-MS/MS. ^k†^Aβ40M; ELISA. ^l†^AβM; binding assay. ^n†^Immune-based detection. ^r†^Aβ40M; liquid phase interaction. ^s†^Aβ42M; binding assay. ^t†^Immune-based detection. ^u†^Aβ42; SPR (K_D_ not reported). ^v†^Aβ42M; pull-down from plasma, WB. ^w†^Aβ40F; dot blots. ^x†^Aβ42M; pull-down, fluorescence polarization. ^y†^Immune-based detection. ^z†^Plaque material spatially targeted optical microproteomics. ^a‡^Aβ42F; immunolabeling, TEM. ^b‡^Aβ40O; NMR. ^c‡^Aβ42M; SPR. ^d‡^Aβ40M; CD titration. ^e‡^Aβ40M; NMR. ^f‡^Aβ42M, Aβ42O; binding assay. ^h‡^Aβ40F, Aβ42F; SPR (K_D_ not reported). ^i‡^Aβ40F; ELISA. ^j‡^Immune-based detection. ^k‡^No significant changes, CSF. ^l‡^Aβ28M, Aβ40M; affinity chromatography. ^n‡^Immune-based detection. ^q‡^Aβ40; dot blots. ^r‡–s‡^Immune-based detection. ^t‡^Aβ40M; incubation, SEC. ^u‡^Aβ40M, Aβ42M; ELISA. ^v‡^No changes, serum and CSF. ^w‡^Aβ40M; ELISA. ^x‡–y‡^Gelsolin used as probe to capture Aβ40/42M/O from rat brain and CSF. ^z‡^Aβ40M; ELISA

### Immunoglobulins

Immunoglobulins were among the first proteins to be identified in senile plaques [26, 27] and it was suggested that AD may be a localized form of immunoglobulin amyloidosis, potentially caused by an antigen that could not be disposed by regular degradation routes. Immunoglobulins constitute a special case among the binding proteins as their natural function is to recognize foreign structures. Hence, it is not clear if their occurrence in the plaque is due to antigen recognition (from the amyloid or some other component) or binding to their constant parts. The fact that the constant part is identified by MS does not exclude that the variable domain is also present but not found the MS analysis.

Auto-antibodies against Aβ were first reported in 1993 [140] and some studies showed that AD patients have lower concentrations of such antibodies [141, 142]. The origin and functional roles of these antibodies are not yet fully understood but they have been heavily investigated with immunotherapy approaches in mind. Interestingly, IgM antibodies purified from AD patients have been found to have catalytic abilities to hydrolyze Aβ and would thereby actively counteract the accumulation of Aβ [143]. The antibodies were demonstrated to inhibit Aβ aggregation as well as toxicity in cell culture.

Moreover, Aβ is not the only antigen for AD-related autoantibodies. Immunoglobulin response against several other proteins/structures, e.g. oxidized low-density lipoprotein, RAGE, and S100b, have also been found [144], potentially reflecting the multiprotein nature of the senile plaques. Recognition of plaque structures by immunoglobulins, either it is the amyloid itself or any other molecular component acting as antigen, can activate inflammatory response and the complement cascade and lead to the effects described below.

### Complement and inflammatory response proteins

Complement activation is critical in normal inflammatory responses to injury and in removing apoptotic cells, tissue debris, and other macromolecular aggregates. Likewise, complement proteins have fundamental roles in the development and protection of the central nervous system. Inappropriate activation of the complement system in the brain may cause neuroinflammation, or even neuronal cell death. Activation of the neuronal complement in AD brain is supported by the identification of several initial and terminal proteins of complement cascade, including complement 1q (C1q) [26, 28, 31, 32, 59, 60, 66, 70, 73], complement 1s (C1s) [32], complement 1r (C1r) [32], complement 3 (C3) [26, 28, 31, 32, 59, 60, 66, 73], complement 4 (C4) [26, 31, 32, 59, 60, 66, 73], complement 5 (C5) [32], complement 7 (C7) [32], and complement factor H [32, 59] in AD plaques. It is likely that the complement system could be activated by Aβ deposition. Indeed, studies demonstrated that Aβ can bind C1q [70, 71], an initiating protein of classical complement activation, and activate the cascade in AD brain in the absence of immunoglobulins [70].

C1q has been found highly increased in human and mouse brains with age [145, 146] and may damage synapses. An adult mouse model lacking C1q (C1qKO) exhibited better synaptic plasticity and significantly less cognitive and memory decline compared to wild-type littermate [145]. In a recent study, soluble Aβ oligomer was injected into C1q deficient mice (C1qaKO) that exhibited no Aβ induced synaptic losses suggesting C1q is required for Aβ induced toxic effect on synapses *in vivo* [146]. Interestingly, C1q has been found to possess a distinct binding site for Aβ [147] and C1q dramatically enhance Aβ aggregation *in vitro* [148]. Hence, the blocking of C1q-Aβ interaction may have potential in AD therapy, which is further supported by the finding that the inhibition of C1q binding to Aβ protects hippocampal cells against Aβ induced complement dysfunction [147].

In addition to C1q, Aβ can bind several other complement proteins *in vitro* [51–53, 56, 72, 75]. Aβ42 aggregates has been found to bind complement proteins such as C1q, C3, C4, C5, and C6 with higher affinity compared to aggregates formed by other Aβ isoforms [72]. Hence, changes in the relative concentrations of Aβ isoforms could play a part in complement activation. Non-fibrillar Aβ has also been found to bind and activate complement components, *e.g.*, C1s and C4, in human plasma in a dose-dependent manner [149]. Aβ-mediated neuronal complement activation may bring toxicity to the neuron cell [150], and thereby contribute to neurodegeneration.

Like C1q, C3 may also damage synapses during the aging process since the protein is found to be increased in the brain and CSF of AD patients as well as mouse models of AD [151]. Indeed, C3 deficient (C3KO) mice did not show age-related synapse loss whereas age-matched C57BL/6J wild type did [152]. Likewise, C3 is also required in AD-related synapse loss as demonstrated using a C3 deleted PS2APP×C3KO mouse model [151]. Interestingly, it has been shown that neuronal Aβ can modulate amyloid pathology through a complement-dependent pathway, in which C3 is a central molecule. Overproduction of neuronal Aβ may activate astrocytic complement pathway, via astroglial nuclear factor kappa B, which subsequently releases C3, this C3 interacts with microglial C3a receptor, thereby impair Aβ phagocytosis [153]. Beneficial roles of C3 in AD pathology are also reported. For instance, aged C3 deficient AD mice (APP; C3(-/-)) showed twofold increased total Aβ and fibrillar plaque burden in cortex and hippocampus compared to aged-match APP transgenic mice with C3 [154]. C3 knockout mice (APP/PS1; C3/KO) also exhibited better performance on a learning and memory task [155].

Alpha 1-antichymotrypsin (ACT) is an inflammatory protein and belongs to the serine protease inhibitor family. Like the complement-related inflammatory proteins, ACT is often found in AD plaques [28, 31, 32, 59, 60, 66, 67]. Moreover, ACT is overexpressed in the AD brain [156], and elevated levels of ACT has been reported in plasma and CSF of AD patients [68, 69, 157], which also correlate well with the severity of the disease [157]. Likewise, the AD risk allele apoE4 is linked to elevated ACT expression. Mice carrying apoE4 showed an increased expression of serpina3 family gene (which coding ACT) compared to apoE2 or apoE3 genotype carriers mouse [158]. Furthermore, the protein has been shown to bind Aβ *in vitro* [50, 63–65] and to promote Aβ fibrillation [65, 159]. Mouse model studies also support the *in vitro* findings. ACT was found to promote Aβ deposition, particularly in hippocampus, in a double ACT/APP transgenic mouse model compared to the mouse that only express APP [160].

In summary, Aβ interacts with several proteins associated with innate immune response and inflammation and could thereby trigger and modulate neuroinflammation, which is a critical part of AD pathogenesis.

### Proteins related to lipid transport and metabolism

Lipids have central roles in cell signaling as well as many physiological processes related to normal brain development and functions [161]. Binding of amyloid aggregates to lipid membranes can affect their integrity [162] and a variety of lipids are found associated with the senile plaques [163, 164]. Brain uptake, metabolism, and utilization of lipids and lipoprotein particles are intimately associated with AD pathology. Two of the apolipoproteins, apoE and apoJ (a.k.a, clusterin; CLU), are associated with AD as major risk genes. The ε4 allele of apoE was early identified as susceptible for late-onset AD [30] and recently two genome-wide association studies have identified CLU variant (rs11136000), which is also associated with late-onset AD [165, 166]. Curiously, apoE has recently been shown to affect AD pathology through its immunomodulatory function which is thought to be associated with its binding to microglial triggering receptor expressed on myeloid cells 2 (TREM2; reviewed in Ref. [167]). Both of these apolipoproteins and many more can directly bind Aβ as discussed below.

ApoE exists in three isoforms—apoE2, apoE3, and apoE4. The protein plays a role in Aβ metabolism and clearance, in which apoE4 is the least efficient variant and thereby represents an increased risk of developing AD [168, 169]. Strittmatter *et al.* [169, 170] reported one of the first lines of evidence for *in vitro* binding of apoE to Aβ and demonstrated that the binding event required residues 12–28 of Aβ. The binding affinity of apoE to Aβ was found to be in the order of apoE2 > apoE3 ≫ apoE4, which inversely correlated with the risk of developing AD [169, 171, 172]. However, the binding efficiency of different isoforms of apoE to Aβ can vary substantially depending on the preparation condition of apoE as well as the species and isoforms of Aβ being used [172]. It has also been shown that apoE can modulate Aβ aggregation *in vitro*, though the actual effect of apoE on Aβ aggregation is contentious. Some of the studies suggest that the binding of apoE to Aβ increases Aβ oligomerization [173], and subsequently promotes its aggregation [159, 174, 175]. In this aspect, apoE4 has been found to be more efficient compared to other isoforms [174]. Mouse model studies also revealed that apoE4 differentially affect Aβ aggregation than other isoforms *in vivo*. A transgenic mouse model expressing apoE4 (E4FAD) showed compact and greater Aβ deposits while apoE2 (E2FAD) and apoE3 (E3FAD) expressing mice exhibited diffuse plaque type [176]. The astrocytic overexpression of apoE4, but not apoE3, suppressed Aβ clearance and also promoted amyloid deposition in cell-type-specific and apoE inducible mouse model [177]. However, the greater effects of apoE4 on Aβ aggregation *in vivo* likely take place during the early stage (seeding stage) of amyloid development [177]. Besides Aβ-associated AD pathology, apoE4 is also found to aggravate tau-mediated AD pathogenesis [178].

Contrary to the aggregation promoting effect, apoE has also been found to have an inhibitory effect on Aβ fibrillation in some studies [179–182]. This inhibitory effect is thought to be directed by the binding of apoE to monomeric Aβ, which results in reduced nucleation and a slower fibrillation process [180, 181]. Furthermore, apoE has been reported to stabilize Aβ oligomers [179, 181] and fibrils [181], though the latter event required a higher concentration of apoE. Nevertheless, it is evident that the apoE-Aβ interaction has direct effects on modulation and clearance of Aβ and hence a key role in the pathogenesis of AD. Therefore, many therapeutic strategies targeting apoE-Aβ interaction have been investigated and some are under way (reviewed in Ref. [183]).

Like apoE, CLU is also a key player in AD pathology and capable of modifying Aβ aggregation. However, CLU may have beneficial roles in AD. The protein has a chaperone function that can specifically inhibit the elongation of Aβ fibrils [184] and it is involved in neural health and Aβ metabolism. CLU has been found to be upregulated in the brain of AD patients [185, 186], and co-deposits with Aβ in the senile plaque [73, 101]. Hence, it may be part of the biological defenses against local damage to neurons, for instance as a consequence of Aβ deposition [185, 187]. *In vitro* studies support this protective mechanism since many studies have shown that CLU can bind Aβ and protect against Aβ aggregation [99, 100, 188, 189] as well as reduce Aβ-associated toxicity [187]. Transgenic mouse model studies also evident that CLU can suppress amyloid formation and reduce amyloid toxicity *in vivo*. The astrocytic overexpression of CLU in APP/PS1 transgenic mouse (APP/PS1^AAV-CLU^) showed a significant reduction of total and fibrillar Aβ in cortex and hippocampus compared to APP/PS1^AAV-GFP^ control [190]. Moreover, CLU overexpression resulted in reduced amyloid-associated neurotoxicity [190]. In support of these findings, the reduction of CLU showed opposite results *i.e.*, substantial increase of amyloid plaque load in both cortex and hippocampus of APP/PS1; Clu^+/−^ mice [190], and in CAA within the cerebrovasculature of APP/PS1; Clu^−/−^ mice [191]. Furthermore, clusterin has been shown to enhance Aβ clearance across the blood-brain-barrier via low density lipoprotein-related protein 2 (LRP2) in C57B16 mice [192], which is in agreement with CLU lacking APP/PS1; Clu^−/−^ mice that showed impaired Aβ clearance [191]. The evidence clearly illustrate the importance of clusterin in AD.

In addition to apoE and CLU, several other apolipoproteins including apoA-I, apoA-II, apoA-IV, apoB-100, apoC-I, apoC-II, apoC-III, apoC-IV, and apoD are in the center of interest in the field of AD research. Accumulation of these proteins in amyloid deposits may disturb the brain lipid metabolism and thereby cause cognitive dysfunction. These proteins can also bind Aβ and modulate its aggregation and toxicity. For example, apoA-I, an abundant plasma protein, can bind Aβ and inhibit its fibrillation [84]. The morphology of pre-formed Aβ aggregates can change when exposed to apoA-I [193] and apoA-I can attenuate Aβ-induced toxicity as demonstrated in two different studies using hippocampal neuronal cells [84, 193]. Not only as individual lipoprotein, but also the high-density lipoproteins (HDL) particles may have inhibitory effects against Aβ fibrillation, as the HDL-complex (containing apoA-I, apoE, and CLU) isolated from CSF of AD patients effectively inhibits Aβ fibrillation *in vitro* [194].

The internalization of Aβ is considered as one of the contributing factors to the toxicity and cell damage in AD. Hence, inhibition of such events may be beneficial against AD. ApoA-II, while forming complex with apoE, appears to have inhibitory effects on Aβ internalization in cell culture [195]. Likewise, apoA-IV may also diminish AD pathogenesis. The genetic reduction of apoA-IV in 5×FAD APP/PS1 transgenic AD mice (5×FAD APP/PS1; apo-IV^−/−^) augments the Aβ burden and aggravates neuronal loss in the brain compared to 5×FAD APP/PS1 transgenic mice with apoA-IV [196]. This mouse model study also illustrated that apoA-IV facilitates Aβ uptake by astrocytes [196]. Besides the direct binding to Aβ, the level of many apolipoproteins in circulation correlates with AD pathogenesis. For instance, the level of apoA-I [87, 89, 92] and apoC-III [92] in plasma, and apoD in CSF [95, 96] were measured to be lower in AD patients compared to non-demented controls, thus suggesting reduced activities of these proteins in lipid metabolism.

Taken together, the close links between the apolipoproteins apoE and CLU, and various aspects of Aβ biochemistry may explain their special recognition as genetic risk factors. Accumulation of these, and other apolipoproteins, in plaques may affect the lipid metabolism and thereby also neuronal function and plasticity.

### Proteins involved in blood coagulation and hemostasis

Vascular dysfunction is commonly observed in AD and may precede onset of the disease [197]. Aβ can deposit and interfere with the vascular cellar milieu, exert toxic effect, induce vascular inflammation, and contribute to vascular pathology. Indeed, Aβ has been shown to bind several key components of the coagulation cascade, including coagulation factor V [50, 51, 56], factor X [51, 52, 109], factor XII [53, 56, 110, 111], and fibrinogen [50–52, 56, 112]. It has been reported that Aβ can bind and activate blood coagulation factor XII that subsequently activates factor XI [111], which in turn promote thrombin generation and lead to the production of unwanted fibrin. This may contribute to neuronal dysfunction in AD by inducing inflammation or by affecting cerebral blood flow [110]. Knockdown of plasma coagulation factor XII in TgCRND8 transgenic mouse (FXII-ASO) showed reduced brain amyloid pathology and improved cognitive function compared to the control group (CTL-ASO) and wild-type mice [198].

Fibrinogen has a central role in the hemostasis process and is also implicated in AD pathogenesis. In a recent study, fibrinogen was shown to induce spine elimination and to promote synapse loss in a 5×FAD transgenic mouse model *via* CD11b-CD18 microglia activation. In contrast, the inhibition of fibrinogen-CD11b binding led to improved cognition in the animals [199]. Fibrinogen induced spine elimination was also noted around Aβ deposits with fibrinogen [199], indicating potential *in vivo* Aβ-fibrinogen interactions. *In vitro*, binding of Aβ to fibrinogen results in the fibrillation of fibrinogen and Aβ itself [112]. Biochemical and structural investigations have revealed the central region of Aβ as crucial for the Aβ-fibrinogen interaction [200]. It is likely that Aβ encounters and binds fibrinogen in the vessel wall, which may lead to CAA and cognitive dysfunctions [113]. Hence, it is evident that Aβ-fibrinogen interactions can lead to neuronal damage and cognitive impairment and may thereby contribute to AD [201].

Plasminogen and antithrombin are parts of negative feedback loops in the coagulation cascade. Both of these proteins can bind Aβ [50, 51, 53, 56, 109] and antithrombin III has also been found in neurofibrillary tangles [108]. Mostly antithrombin acts on thrombin and reduces the amount of thrombin to avoid continuous or excess fibrin production. Antithrombin-III has shown sub-micromolar affinity to Aβ [50] and binding to Aβ may result in loss-of-function effects of antithrombin-III. Indeed, excess thrombin production is reported in the AD brain [202]. However, high CSF levels of antithrombin-III have been reported in the early stage AD [69, 80].

Alpha-1-antitrypsin (A1AT) and alpha-2-macroglobulin (α2M) can modulate inflammation, proteostasis, and apoptosis. Both proteins show potential as AD biomarkers [61, 76, 89, 102, 104, 107], can interact with Aβ [51–53, 56] and may have beneficial roles in AD. For instance, A1AT has been shown to protect primary microglial cells isolated from Swiss Webster mouse embryos from Aβ induced toxicity [203], and α2M can prevent Aβ fibrillation *in vitro* [105, 204]. Moreover, α2M has been suggested to facilitate brain Aβ clearance [105, 106].

Hence, co-aggregation of Aβ and blood coagulations proteins may contribute to CAA development and thereby enhance the cognitive deficiencies. However, disturbances of the hemostasis machinery could also impede the coagulation cascade and lead to an increased risk of micro hemorrhage, which is also reported for AD patients [205].

### Proteins involved in metabolism

Although the proteins in this category have their main roles intracellularly, they can also be secreated or located to the cell surface. Their extracellular concentrations may also increase as result of neuronal death and lysis [206]. Dysfunctional glucose metabolism is thought to play a critical role in the pathogenesis of AD. Factors that contribute to the impaired glycolysis include over-use (to compensate for increased demand in AD condition), inhibition and damage (due to oxidative stress or unwanted binding to other proteins such as Aβ) of glycolytic enzymes. Among the glycolytic enzymes, glyceraldehyde-3-phosphate dehydrogenase (GAPDH) and enolase are frequently found in senile plaques.

GAPDH is an enzyme, which in its modified forms (*e.g.*, oxidation and glycation) can interact with several molecular partners that are associated with normal cellular as well as pathogenic functions [207]. GAPDH can interact with monomeric [50, 63] and fibrillar [51, 56, 116] forms of Aβ and tau [208]. Such interactions may contribute to reduced activity or even inactivation of GAPDH. Indeed, it has been found that the GAPDH activity in AD brain is low and triggered by oxidative stress [207]. The fact that GAPDH was found to bind Aβ fibrils in biological fluids shows that the plaque-associated GAPDH can originate from secreted GAPDH. Although the main functional roles of this protein are found in the intracellular environment it can also be secreted [209], a process that may be associated with cellular iron imbalance [210]. Moreover, upon exposure to oxidative stress, GAPDH can form amyloid-like fibrils that contribute to neuronal cell death [211, 212], possibly by gaining toxic function. Aggregated GAPDH has also been shown to accelerate Aβ fibrillation *in vitro* and GAPDH, when co-administered with Aβ into C57BL/6J mice, showed increased Aβ induced neurotoxicity compared to the mice that were treated with either Aβ or GAPDH alone [213].

Alpha-enolase (ENO1) is expressed on the surface of several cell types, including neurons, and acts as a receptor and activator of plasminogen [214]. It has been identified in senile plaques in several studies [32, 59, 60, 66, 115] and been found to be up-regulated in the brain of AD patients [215] as well as in the brain of aged Tg2676 AD mouse model [216]. ENO1 is also oxidatively modified in the progression from mild cognitive impairment to AD [217]. Indeed, a mouse model study showed that ENO1 is a target of oxidation in the brain of 3×Tg AD mouse when compared to non-transgenic mouse [218]. This suggests that beside the glucose metabolism, ENO1 may play critical roles in pathological brain functions. The physiological impact of the Aβ-ENO1 interactions is unknown. However, binding of Aβ to ENO1 may inactivate the enzyme resulting in loss-of-function effects and thereby contribute to glucose hypometabolism in the AD brain [217].

Procollagen C-endopeptidase enhancer 1 (PCPE1) is a glycoprotein involved in procollagen processing. In addition to its role in procollagens maturation, PCPE1 is thought to have additional functions [219] reinforced by its ability to interact with several proteins. PCPE1 is found to form a complex with Aβ [219], but the consequences are not known. Curiously, PCPE1 is reported to initiate amyloid fibril formation of β2-microglobulin [220]. Phosphoglycerate kinase 1 (PGK1) is another glycolytic enzyme, which also binds Aβ *in vitro* [50, 56] and co-deposits with Aβ in plaques [31, 32, 59, 60, 66]. Nevertheless, the role of these proteins in AD has not been investigated.

Binding of metabolic enzymes to amyloid plaques could certainly disturbed their functions and reduce neuron viability. It is, however, not clear how sequestration of extracellular enzymes would affect intracellular processes. The GAPDH and ENO1 examples illustrate that these proteins may have other biological functions that are yet not fully understood.

### Molecular transport proteins

Alteration or interruption of the transport pathways for nutrition and essential biomolecules can have adverse effects on cell viability. Several proteins with transport as main functions have been found in post-mortem plaques and their binding to Aβ have been confirmed by *in vitro* studies.

Hemoglobin (Hb) is the major transporter of oxygen to the body tissues, including brain tissue. In brain, Hb is expressed in specific cells, such as neurons [221, 222], and the level of Hb goes up during aging. Likewise, endogenous Hb is elevated in the brain in response to hypoxia [223]. Hb from the circulation may also contribute to the higher Hb level in the brain due to the changes in the blood-brain-barrier structure, which is manifested in aging as well as in AD. Hb has been found to be up-regulated in the brain of APP/PS1 transgenic mice compared to wild-type littermates [224] and in the brain of AD patients [225]. The protein colocalizes with Aβ in plaques and vascular amyloid deposits [63, 226]. Hb can bind Aβ *in vitro* and promote its aggregation [224, 226]. Hence, in aging or AD, the oxygen deficiency in brain may lead to an increased Hb production, which in turns may modulate Aβ aggregation or clearance in the brain.

Hb degradation can lead to release of heme and redox-active iron that can trigger the formation of reactive oxygen species and oxidative stress. Indeed, dysfunctional iron homeostasis is part of the AD pathology [227, 228]. Heme can also bind directly to Aβ [229, 230] with resulting peroxidase activity of the complex [231, 232]. This process could further enhance the oxidative stress and potentially also affect Aβ assembly by catalyzing tyrosine crosslinking of Aβ molecules [233].

Protection against the release of heme is achieved through the proteins haptoglobin (Hpt) and hemopexin (Hpx), that are both found to be binding partners of Aβ fibrils. Hpt is well known for binding free hemoglobin after the intravascular hemolysis thereby preventing iron loss [234] while Hpx is a scavenger for free heme groups. Hpx-null (Hpx^−/−^) mice showed a twofold increase in iron-loaded oligodendrocytes in the basal ganglia and thalamus compared to wild-type mice, which verified that Hpx is involved in heme scavenging in brain [235]. Hpt appears to act as an extracellular chaperon and counteract protein aggregation [236]. The Aβ-Hpt complex is found in brain tissue and CSF of AD patients and the interaction is confirmed by *in vitro* data [237]. The binding affinity (K_D_) is 0.30 μM [237]. Moreover, Hpt can potently inhibit *in vitro* Aβ fibrillation [204] and Aβ clearance by enhancing apoE-Aβ complex formation [237]. Hpt as well as Hpx are acute phase proteins that associate with lipoparticles [238], which connect them to inflammatory processes and lipid metabolism described above.

Serum albumin is abundant in both plasma and CSF where it binds and transports a range of different molecules, including Aβ [239]. The protein is thought to bind ca. 90% of plasma and 50% of CSF Aβ [240] and may act as a potent inhibitor of Aβ self-assembly in the circulation. *In vitro* studies showed that albumin inhibits Aβ fibrillation by binding to the monomer [239], or by binding to Aβ oligomers and compete for further monomer association to the Aβ assemblies [241, 242]. *In vivo* studies corroborates *in vitro* finding as a 3×Tg mouse model treated with human serum albumin exhibit reduced Aβ deposition and ameliorated cognitive impairment. Moreover, SHSY5Y cell line treated with human serum albumin resulted in a reduction in Aβ toxicity [243]. Notably, low levels of blood albumin in elderly persons is implicated in cognitive impairment [244], and evidently decreased blood albumin-Aβ complex is found in AD [245]. In addition, albumin is an attractive target for enhanced brain Aβ clearance through the exchange of plasma albumin with therapeutic albumin molecules, which may also facilitate the efflux of Aβ from brain to plasma [117, 118]. A pilot plasma exchange (PE) study for mild to moderate AD patients reported stable cognitive scores, and in phase II clinical trial, the patients were found to perform better in the cognitive test [246]. More recently, results from phase IIb/III clinical trials have been published [247], and these results also suggest that PE treatment could slow cognitive and functional decline in AD.

Transthyretin (TTR) is a tetrameric protein predominantly produced in the liver and functions as a carrier of thyroxine and retinol in plasma and CSF [248]. TTR binds Aβ with high affinity, K_D_ of 28 nM [121]. The binding site of Aβ on the TTR monomer appears to be residues 106–117 [249] and *in vitro* studies showed that TTR can inhibit Aβ fibrillation in buffer and CSF [121, 250]. TTR-Aβ complex has been detected in CSF [250], and TTR has been suggested as a major Aβ-sequestering protein in CSF [251]. The protein has also been shown to promote Aβ clearance and reduced deposition in the brain of AβPPswe/PS1A246E transgenic mice carrying TTR [252]. Moreover, the overexpression of human TTR in APP23 transgenic mice (APP23/hTTR) showed improved cognitive function compared to control APP23 mice [122]. The TTR concentration in CSF is altered in the course of AD [253] and TTR has therefore been suggested to play protective roles in AD. The protective mechanism likely relies on the binding of TTR to oligomeric Aβ, which inhibits primary and secondary nucleation of Aβ and thereby limits Aβ fibrillation [254].

Other transporter proteins such as inter-alpha-trypsin inhibitor heavy chain H2 (ITIH2) and serotransferrin are found in senile plaques [31, 32, 59, 66]. ITIH2 is a carrier of hyaluronan, whose increased level is associated with AD [255]. Serotransferrin is involved in iron transport and abnormal iron metabolism is, as already mentioned, observed in the brain of AD patients and can also influence Aβ aggregation [256].

Taken together, the described examples highlight two classes of transport proteins that may have key roles in AD pathology: transporters of Aβ could affect its clearance and accumulation while dysfunctional iron transporters are linked to oxidative stress and neuronal damage.

### Neural proteins

Neurosecretory protein VGF is a nerve growth factor that regulates neuronal development and activity through processing of the precursor protein into bioactive peptides. One such peptide, TLQP-21, has been shown to enhance Aβ clearance through microglial phagocytosis and to promote fibrillar Aβ uptake by microglial BV2 cells through a complement C3a receptor-1 (C3aR1)-dependent mechanism [257]. A more recent study further demonstrated TLQP-21 mediated microglial modulation via C3aR1 using wild-type and C3aR1-null mouse models [258]. TLQP-21 was found to increase motility and phagocytic activity of microglial BV2 cells in wild-type but not in C3aR1-null mice. Furthermore, intracerebroventricular administration of TLQP-21 to 5 months old 5×FAD mice showed a reduction of amyloid plaques and associated dystrophic neurites [258]. However, the levels of VGF in AD patient CSF has been found to be lower than in controls [79, 90, 124, 125]. A recent bioinformatics study identified VGF as the “key driver” in a multiscale network model of AD [259]. The same study validated the hypothesis by showing that overexpression of VGF in an AD mouse model rescued the animals from Aβ-related pathology. Based on these lines of arguments, sequestration and inactivation of VGF in plaques may be a key process in AD-associated neurodegeneration.

Like VGF, proSAAS is a proteolytically processed protein with the main function in the neuroendocrine secretory pathway [260]. It is recurrently found in pathological protein deposits [261–263] and several studies have identified it as a potential biomarker of AD [62, 78]. ProSAAS has been found highly colocalized with Aβ, both in dense core and diffuse plaques, in 12-month-old APP695/PSEN1dE9 transgenic mice [264]. ProSAAS is thought to have novel anti-aggregation chaperone function demonstrated by its ability to inhibit *in vitro* Aβ fibrillation and Aβ-induced neurotoxicity in Neuro2a cell cultures [264]. Furthermore, proSAAS has also been found to inhibit fibrillation and toxicity of other disease-related proteins *e.g.*, IAPP and α-synuclein [265, 266].

Neural cell adhesion molecule 1 (NCAM1) is an important component of the central nervous system (CNS) extracellular interface but also found in soluble form in CSF and plasma. NCAM1 has key roles in modulation neuron-neuron adhesion, neurite outgrowth, synaptic plasticity, as well as in learning and memory and is also associated with several neurological disorders [267]. It interacts with a range of other proteins and extracellular matrix (ECM) components, including APP [268] and chondroitin sulfate proteoglycans (see below) [269]. Another member in the NCAM superfamily, NCAM2, has been shown to undergo Aβ-induced proteolysis resulting in reduced level of NCAM2 in hippocampus of AD patients and APP23 transgenic mice [270].

Brevican and neurocan are chondroitin sulfate proteoglycans specifically expressed in the brain and neural tissues. They are involved in CNS development, cell migration, maturation, and tissue homeostasis and are key components of perineuronal nets [271]. It is known that altered regulation of these proteoglycans, e.g. by ADAMTS family proteases, is associated with AD as well as inflammation and other neurological disorders [272]. Aβ accumulation may interfere with processing of brevican and thereby inhibit neural plasticity [273]. Indeed, brevican has been found differently processed (the size of the chondroitin sulfate chain attached to brevican is smaller) in hippocampal tissue of plaque-bearing APPsw transgenic mice compared to non-transgenic control [273].

Amyloid-like protein 1 (APLP1) is a transmembrane protein, which belongs to the amyloid precursor protein gene family. Members of this family are known to play critical roles in the development of nervous system, the formation and function of synapses, including synaptic plasticity, learning, and memory [274]. APLP1 is closely related to APP and share a similar structural organization [275]. It is processed by the same set of secretases *i.e.*, α–,β–,γ–secretase, as APP and generates a range of fragments [276], but not Aβ [274]. Immunochemistry and proteomics studies show that APLP1 is present in the senile plaque and the distribution of APLP1 overlaps with APP in the AD brain [32, 59, 277, 278]. APLP1 binds fibrillar Aβ *in vitro* [51, 52, 56], although the pathological significance of the interaction is not known. Nevertheless, the binding of fibrillar Aβ to APLP2, from the same family as APLP1, lead to an increased level of APLP2 in primary cultures of astrocytes and neuron [279].

The accumulation of neural proteins in plaques provides a direct link between protein aggregation and reduced neuronal functions. Some of the proteins may also have neuroprotective effects that are lost upon their inactivation.

### Cell adhesion, extracellular matrix, and proteoglycans

The extracellular matrix (ECM) proteins and proteoglycans are often found in senile plaques and it has been confirmed that Aβ, and in particular its aggregated forms, can bind a range of ECM-associated proteins [109]. Interactions with these components provide a link between extracellular environment, where the senile plaques are found, and potential intracellular effects. This could be by triggering specific signaling pathways or by allowing entrance of foreign pathogens, such as amyloid oligomers. Proteins such as vitronectin may assist in this [280]. The ECM also determines the contact and interactions between adjacent cells and correct function is crucial for many processes, such as neuronal development and signaling pathways.

Proteoglycans are thought to play functional roles in cell-cell and cell-matrix interactions in the brain. Moreover, proteoglycans appear to modulate Aβ aggregation and deposition and to play roles in Aβ internalization and cytotoxicity [281–283]. A transgenic mouse model study showed that the overexpression of heparanase, a heparan sulfate degrading enzyme, significantly lowered the Aβ burden in the brain of tgHpa*Swe mice [282]. In a different study, neuronal heparan sulfate was removed from APP/PS1 transgenic mouse, which led to a reduction of Aβ oligomerization and subsequent deposition [283]. Also, enhanced Aβ clearance from the mouse brain was noted [283]. These studies evident that heparan sulfate proteoglycans participate in Aβ deposition and thereby contribute to amyloid pathology.

Agrin is a heparan sulphate proteoglycan present in different areas of the brain including microvasculature but its functions in the brain remains unclear [284]. The protein has been identified in AD plaque, cerebrovascular Aβ deposits as well as in neurofibrillary tangles in several studies [32, 59, 60, 66, 126, 284]. *In vitro* studies reported that agrin can bind Aβ and accelerate its fibrillation as well as protect fibrils from proteolytic degradation [126]. Curiously, a mouse model study reports opposite effects of agrin on *in vivo* Aβ deposition. Endothelial overexpression of agrin in AD transgenic mouse resulted in reduced Aβ accumulation, and conversely, mouse lacking endothelial expression of agrin showed an increased Aβ deposition in the brain [285]. However, it is apparent that agrin affects brain Aβ deposition. Agrin may have a specific affinity towards fibrillar Aβ (K_D_ of 3.5 nM [51]) compared to other forms of Aβ aggregates and monomer [109, 126]. Increased concentration of agrin has been measured in the hippocampus of AD brains compared to non-AD controls [286]. Moreover, CSF agrin concentration increased with the age of AD patients [51, 127], which indicates a possible link with the disease progression. Agrin abnormalities are also thought to cause microvascular damage in AD [286].

Likewise, glypican-1 (Gpc-1) seems to be predominant in senile plaques [287] and cerebrovascular amyloid deposits [288] in AD patients. Notably, Gpc-1 is not present in the normal vessels [289], indicating Aβ may modulate the cellular expression of Gpc-1. Indeed, it has been found that the expression of Gpc-1 is increased in human brain pericytes culture in the presence of Aβ [289]. Within lipid rafts, Gpc-1 is thought to act as a scaffold and interact with normal cellular and disease-associated isoforms of prion protein and facilitate misfolding, thereby formation of pathogenetic prion [290]. Such mechanisms may also occur for Aβ pathogenesis. Aβ production machineries, as well as monomeric and oligomeric Aβ, exist in glycosphingolipid enriched domains where Gpc-1 may interact with Aβ and potentially trigger Aβ polymerization and subsequent cell death [130].

Yet another proteoglycan family protein, decorin, is also found in senile plaques [32, 129] and neurofibrillary tangles in AD patients [129]. The protein can interact with Aβ *in vitro* [50, 51, 56, 109, 128]. However, the significance of this interaction *in vivo* has not been investigated broadly although the interaction likely also occurs in the AD brain. The binding of decorin to Aβ may account for the deposition of Aβ in cerebrovascular amyloid deposit in AD [128].

Cartilage acidic protein 1 (CRTAC1), an extracellular matrix protein in the β-propeller protein family, is present in the brain, but its function remains unclear [291]. Nevertheless, CRTAC1 is linked to many diseases, including cardiovascular and neurological disorders [291]. Curiously, the protein has a high propensity to form amyloid-like fibrils that may connect it to disease pathology [292], but this aspect is poorly described. Vitronectin is another cell adhesion protein with the ability to form oligomers and amyloid fibrils [293]. It was early reported to be part of senile plaques [28] and its role in AD pathology may be related to its modulation of complement activation.

Osteopontin (OPN), a protein with its main role in bone tissue structure, has been confirmed to be elevated in AD patients and there are findings that link the OPN expression to the Aβ load [294]. It is linked to immune response and neuroinflammation [295, 296] and may also have a role in calcification of vessels in CAA [297]. Increased cerebral expression of OPN (*via* glatiramer acetate (GA) or GA- and bone marrow-derived monocytes treatments) resulted in reduced plaque burden in ADtg mice compared to mice treated with phosphate buffer. These mice also showed improved phagocytotic clearance of Aβ [298].

Galectin-3-binding protein (G3BP) was just recently shown to inhibit the processing of APP into Aβ by direct interaction with APP [299]. Co-deposition of G3BP in senile plaque may be a consequence of this interaction but it could also result in reduced inhibitory capacity and thereby a positive feedback loop for Aβ production. Direct interaction with APP has also been reported for fibulin-1 with consequent alterations of the neurotrophic activities of APP [300]. This interaction was suggested to be mediated by the calcium-binding EGF motif in fibulin-1 and could then explain also the binding of EGF-containing fibulin-like extracellular matrix protein 1 to Aβ.

Some cell adhesion proteins, *e.g*. vitronectin, osteopontin, fibulin-1 and galectin-3-binding protein, are known to also regulate complement system, immune response, inflammatory response or hemostasis and could thereby contribute to AD pathology through these processes [280, 295, 296, 301–303]. As seen in Table 1, there are several other proteins in this functional category, such as collagen alpha-1(XVIII) chain, desmoplakin, microfibril-associated glycoprotein 4, mimecan, prolargin, and SPARC-like protein, that are found in senile plaque, and with Aβ interactions demonstrated *in vitro*. However, the physiological significance of these proteins in AD still needs to be elucidated.

In summary, it is not surprising that many ECM- or cell-surface proteins are found in plaques but several of these proteins appear to be actively involved in the initiation and buildup of Aβ aggregates. A better understanding of these interactions may reveal ways to delay the plaque pathology.

### Other proteins

In addition to the proteins reviewed above, several proteins with confirmed binding to Aβ as well as presence in senile plaques, fall outside the functional categories in Table 1. Human cystatin C (CysC) was originally identified in CSF and subsequently found in other biofluids and tissues, including brain tissue [304, 305]. The association of CysC with AD has been established by its existence within parenchymal and vascular amyloid deposits in AD [31, 32, 59, 60, 66, 306]. Evidence suggests that CysC plays protective roles in AD. It can bind monomeric Aβ [307], significantly reduce Aβ oligomerization [132] and subsequent fibrillation [133, 307]. Moreover, CysC has been shown to protect neuroblastoma cells from Aβ induced neurotoxicity [308]. A mouse model study showed *ca.* 50% decline of Aβ plaque burden in the cortex of Cys68^+^APP23^+^ transgenic mice overexpressing human CysC compared to Cys68^−^APP23^+^ mice [307]. Besides its protective roles, CysC is also implicated in the development of CAA, and its binding with Aβ may initiate Aβ accumulation in vessel walls [306]. Apart from its association with AD, CysC itself form amyloid and is linked to hereditary cystatin C amyloid angiopathy, or Icelandic type amyloidosis [309].

Gelsolin is a ubiquitous actin-binding protein present in plasma and CSF as a secretory protein [310, 311]. The protein is linked to a Finish type of systemic amyloidosis (hereditary gelsolin amyloidosis) [312] in which amyloid deposition of abnormally processed gelsolin leads to cranial and sensory peripheral neuropathy, corneal lattice dystrophy, and cutis laxa [312, 313]. Gelsolin has been found in AD plaques [31, 32, 59, 60, 66], indicating its potential networking with Aβ, which is reinforced by several lines of *in vitro* studies [50–53, 56, 135]. Moreover, gelsolin inhibits Aβ fibrillation and dissociate preformed fibrils *in vitro* [314]. Peripheral administration of gelsolin (from bovine plasma) in PS/APP transgenic mice showed reduced fibrillar Aβ in cerebral cortex and hippocampus [315]. Another study also reported reduced amyloid pathology in mice where human gelsolin was expressed in plasma in two different PS/APP transgenic mice models using a gene delivery approach [316]. These studies indicate that gelsolin may sequester plasma Aβ and thereby represent a candidate for a potential AD therapeutic.

Serum amyloid P component (SAP) is an important regulator of the innate immune system. Nonfibrillar SAP is a universal component of all amyloid deposits, including parenchymal and vascular Aβ deposit in AD [28, 32, 59, 60, 66, 317–319]. The role of SAP in amyloidosis has been studied using SAP knockout (SAP^−/−^) serum amyloid A amyloidosis mouse. Amyloid deposition in mice lacking SAP was found to be delayed and reduced compared to (SAP^+/−^) control [320]. However, if and how SAP may contribute to AD pathology remains unclear. Interestingly, the protein has a high binding affinity for Aβ, with a K_D_ of 6 nM [139]. Evidence from *in vitro* studies suggests that SAP accelerates Aβ fibrillation and stabilizes fibrils against proteolytic degradation [139, 321, 322]. SAP may also modulate astrocytic and microglial Aβ uptakes as the ability of adult human astrocytes and microglia to internalize Aβ were found to be reduced when exposed to Aβ and a mixture of SAP-C1q [323]. Besides its role in amyloid deposits, SAP is itself directly toxic to neurons [324, 325]. SAP can be an attractive therapeutic target for AD. Indeed, it has been demonstrated that removal of SAP (using CPHPC, a SAP removal drug) from blood and CSF of a TASTPM transgenic mouse model of AD results in removal of it from the amyloid deposits, although the actual effect of CPHPC treatment on amyloid load was not investigated [326].

Secreted frizzled-related protein 3 is involved in the Wnt signaling pathway [327]. This pathway is fundamental to the development of the CNS and, in the adult brain, it is involved in the regulation of synapse plasticity and memory progress [328]. Using neuronal cell culture and an animal model, it was shown that Aβ fibril-mediated toxicity can result in loss-of-function of Wnt signaling [329]. Dickkopf-related protein 3 is also a Wnt signaling pathway protein. The protein has been found to be produced locally and co-localized with Aβ in the brain [134]. Several *in vitro* studies have demonstrated its binding to Aβ [51, 52, 56], and it may have a particular affinity to fibrillar Aβ, to which it showed 26 nM affinity, over oligomers [51].

Other proteins with fundamental roles in early development (*e.g.*, olfactomedin-like protein 3), cell mobility (*e.g.*, cytoplasmic actin 1), and antimicrobial and proteolytic activity (*e.g.*, dermcidin) are found in senile plaques and bound to Aβ *in vitro*, nevertheless, their links with AD is still scarcely described in the literature.

Finally, we want to briefly mention two important proteins that are not included in Table 1 due to the employed selection criteria: α-synculein and tau. The roles of these proteins in AD have been extensively described in other reviews, *e.g.* [330, 331]. Although these are primarily intracellular proteins they are also found in CSF. α-synculein was early associated with plaques as the origin of the non amyloid-β component (NAC) [332, 333] but is better known as the key player in synucleinopathies, including Parkinson’s disease, dementia with Lewy bodies and multiple system atrophy [334]. Lewy body co-pathology is indeed common in AD and a more extensive role of α-synculein in AD pathology is emerging [330]. In vitro experiments have confirmed interactions between Aβ and α-synculein but none of the studies using biological fluids (that constitute the basis for Table 1) identified the protein. This indicates that the origin of plaque-associated α-synculein (NAC) may be intracellular (*vide infra*). Tau has already been mentioned as the main component in neurofibrillary tangles but it has also been found in plaques [31–33, 59]. This constitute one (of many) direct connection between amyloid and tangle pathologies although the role of plaque-associated tau in AD is most likely minor to that of its intracellular accumulation. Despite the fact that an increased tau level in CSF as an established AD biomarker, tau was not identified as a binder to Aβ in our studies with CSF samples from AD patients [50, 51, 56]. Hence, like in the case with α-synculein, tau in the plaques may originate from released intracellular aggregates.

## Network analysis

**Fig. 3:**
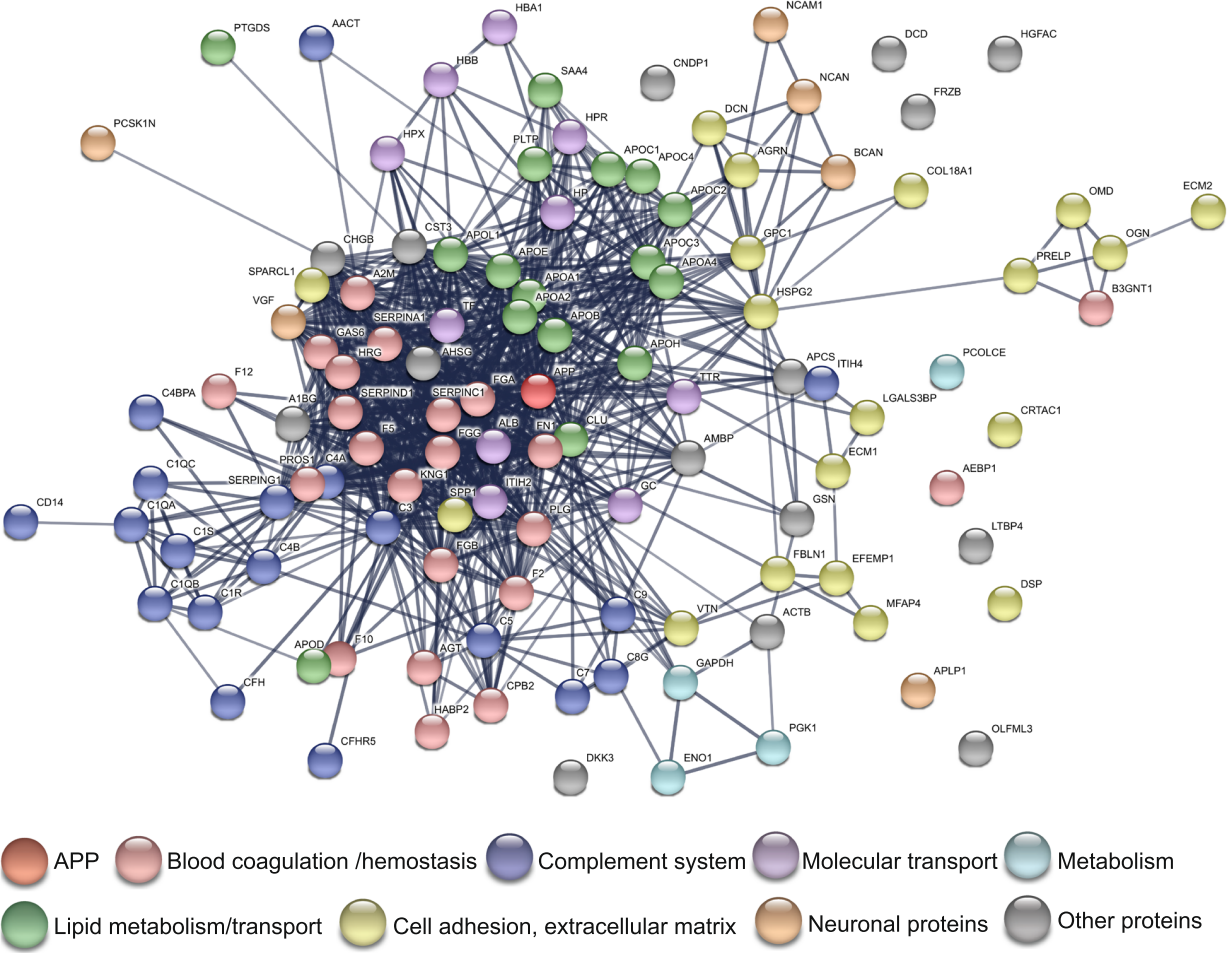
STRING protein network analysis of the proteins in Table 1 (except immunoglobulins). The nodes are colored based on the functional classification used in the article and the thickness of the connecting lines show the confidence of the association

**Fig. 4:**
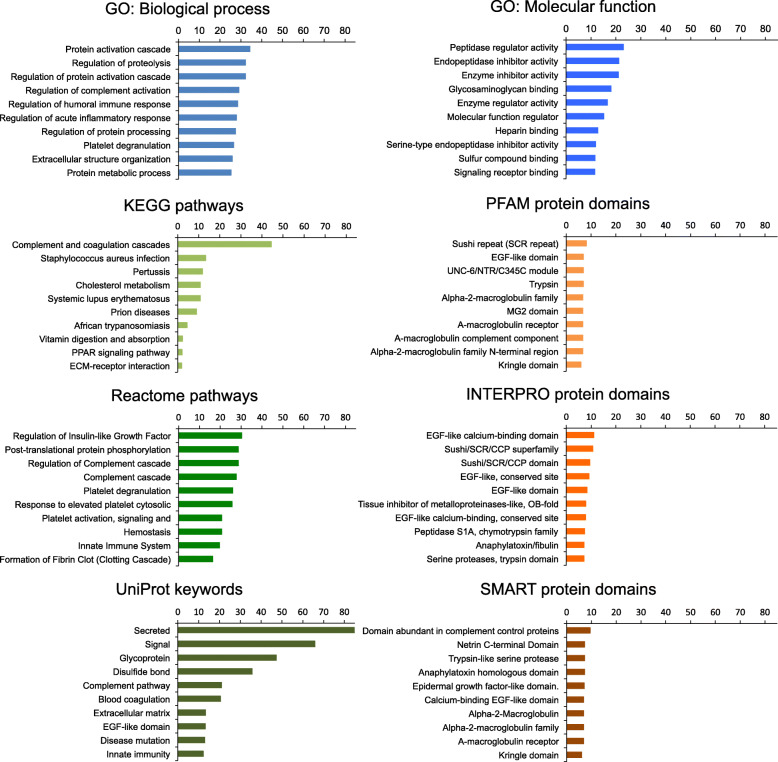
Functional and structural enrichment in the STRING network. The bar diagrams show the –*log*_*10*_ of the false discovery rate for the 10 most enriched keywords from each category

In order to gain new insights from the assembled list of proteins we analyzed it (with the immunoglobulins excluded) by the STRING protein network tool [335]. The network obtained with the ‘high confidence’ setting is illustrated in Fig. 3. There is indeed a dense network of connections between the proteins on the list and we find Aβ (APP) in a central position. The functional classes used in Table 1 are rather well reflected also in the network illustration. It seems that proteins involved in lipid metabolism or blood coagulation in general are found closer to APP than the other classes. The majority of the nodes without any connection are classified as “other proteins”. STRING analysis also provides information about enriched properties of the network (Fig. 4). We note that characteristics related to various protein functions seem to be more enriched in the network than structural features. Hence, the protein composition of the plaques may be connected to the functional roles of the proteins rather than their structural and chemical properties. This is in line with absence of clear correlations between the binding to Aβ fibrils and physicochemical properties observed in previous studies [53, 56]. This means that the network may provide information about biological processes that can be triggered or inhibited by Aβ amyloid. The processes that are highlighted as enriched in this network include protein activation/regulation/metabolism (and thereby various signaling pathways), complement system, blood coagulation, inflammation and immune response. Interestingly the second most enriched *KEGG pathway* is “Staphylococcus aureus infection”, which may indicate that Aβ fibrils could trigger responses similar to bacteria infection.

### Intracellular proteins

**Fig. 5:**
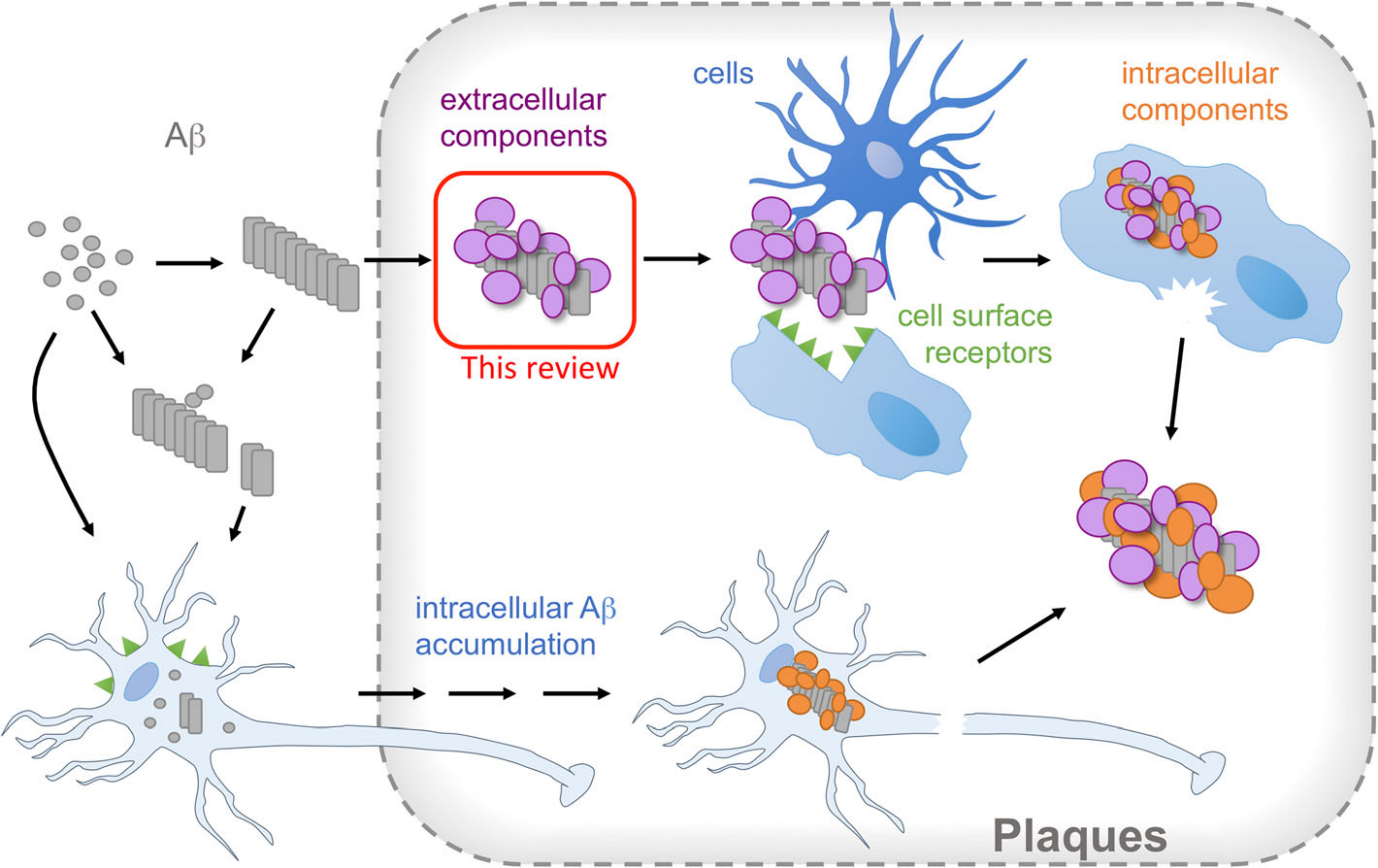
Illustration of how extracellular and intracellular components may end up in senile plaques. Most of the produced Aβ are secreted and can under certain circumstances aggregate in the extracellular environment. The amyloid will bind to extracellular components to form multicomponent aggregates. Amyloid deposits can also trigger cellular response through cell surface receptors leading to phagocytosis and co-accumulation of intracellular components. In another route, the extracellular amyloid can promote the formation of oligomeric Aβ. These oligomers, as well as Aβ monomers, can be internalized by cells and accumulate in the intracellular environment. Too high intracellular load of aggregated proteins may eventually lead to cell death and release of the aggregated material

Although Aβ amyloid plaques are per definition extracellular inclusions, investigations of plaques from brain tissue have revealed the presence of a large number of intracellular proteins. These are clearly parts of the plaques as they have been found by top-down studies of tissue [31–33] and Fig. 5 illustrates potential pathways by which intracellular components could associate with the plaques. Aβ is produced in different cellular locations. Some release may occur directly from the plasma membrane [336, 337] but the main production happens in the endosomal compartment or trans-Golgi network followed by vesicle recycling into the extracellular space [338], where it, under certain circumstances, may begin to aggregate. The formed amyloid will rapidly associate with other extracellular components, as outlined in the present review. The amyloid plaques can activate the cellular defense and inflammation, mainly microglia but also astrocytes [339–341], that can bind to amyloid through various cell surface receptors *e.g.*, Toll-like receptors (TLRs), scavenger receptors such as CD36 and TREM2 [342–345]. The plaques can then be phagocytosed with the lysosomal degradation machinery as destination. In this state the amyloid structure may associate with intracellular components. However, too high load of engulfed material could lead to lysosomal dysfunction and release of the aggregated material to the extracellular space [346].

An alternative route involves intracellular Aβ accumulation (Fig. 5). Aβ is produced in intracellular locations, including the trans-Golgi network, endoplasmic reticulum (ER), and endosomal, lysosomal and mitochondrial membranes [336]. Moreover, neurons (as well as other CNS cells) can actively take up Aβ (monomeric or aggregated) from the surrounding through a number of cell surface receptors, including the α7 nicotinic acetylcholine receptor (α7nAChR), low-density lipoprotein receptors (LDLR), the scavenger receptor for advanced glycation end products (RAGE), and N-methyl-D-aspartate (NMDA) receptors [336, 347–350]. Several reports have described intra-neuronal aggregation and amyloid formation of Aβ (*e.g.*, [351–354]). This process may eventually kill the cells and release the amyloid with the sequestered intracellular components [206]. Taken together, all the material indicated in Fig. 5 could be considered as potential components of the plaques, including Aβ amyloid with sequestered extra- as well as intracellular components, activated cells attached by the surface receptors, intact cells with intracellular amyloid aggregates and debris from dead (and lysed) cells.

*In vitro* biochemical binding studies of intracellular proteins similar to those in biological fluids are few. Olschza *et al*. reported some proteins interacting with Aβ in human embryonic kidney 293 (HEK293T) cells, including translation initiation factors, chromatin regulators, RNA processing proteins, mitochondrial membrane proteins and chaperones. It is not clear, however, what form(s) of Aβ that was responsible for the interactions [45]. Another study used murine neuroblastoma N2a cells and investigated the binding of proteins to Aβ42 oligomers [355]. They found ribosomal proteins, chaperones, and proteins associated with cytoskeleton or protein synthesis. Experimental protocols to identify interaction partners from an intracellular environment are indeed more challenging than in biological fluids but could also provide extremely valuable information, *e.g.*, from comparison of different cell types or even subcellular compartment.

## Conclusions

Just by recognizing the normal and potential abnormal functions of the proteins listed in Table 1 it is evident that many of the proposed pathological mechanisms of AD are covered. This picture is also confirmed by the network analysis. With this in mind it appears unlikely that sequestration of biomolecules in the plaques is merely a side effect of Aβ aggregation and Aβ-mediated toxicity. More likely, co-aggregation and alteration of the associated biochemical processes are part of the mechanisms by which amyloid formation leads to neurodegeneration. However, it still remains an open discussion which of the processes that are more important and how they may affect each other. To address these questions it is important to gain better understanding of the molecular events (how do the proteins bind to the plaque components and how does the binding affect their structure and function) as well as the physiological consequences. Hence, the multicomponent structures of senile plaques should be explored with *both* bottom-up and top-down approaches.

Among the outstanding questions regarding the molecular perspective are to decipher the details of the architecture of the multicomponent plaques; are they just random aggregates of sequestered proteins or is there some kind of order? *In vitro* binding studies suggest that many proteins can bind Aβ with K_D_ in the μM-nM range, which suggest specific binding event rather than unspecific sequestration. Within the network there are also many proteins where mutual interactions are part of their biological function. Such interactions could be important in recruiting certain proteins to the plaques.

More knowledge is also needed regarding whether the bound proteins retain their native structure or if the interaction can lead to misfolding. It is intriguing that many of the proteins that associate with senile plaques *in vivo* or plaque-like particles *in vitro* are known to form amyloid themselves and to be associated with amyloid diseases. This group of proteins include immunoglobulins, apolipoproteins (A-I, AII, A-IV, C-II, C-III), serum amyloid A, fibrinogen, cystatin C, gelsolin, and transthyretin. Notably, NAC (*i.e.* α-synuclein) [332, 333] is not found in Table 1. There are also proteins with confirmed ability to form amyloid aggregates but without any (known) connection to disease, e.g. GAPDH [211, 212], cartilage acidic protein 1 [292] and vitronectin [293]. So far nothing is known about their conformational status when incorporated in the plaques.

How is further Aβ aggregation affected by the binding of other proteins? As described above many of the sequestered proteins are known to affect Aβ aggregation *in vitro*. Moreover, surface-catalyzed secondary nucleation seems to be a critical part in the amplification of Aβ aggregates [356] and that process will certainly be affected by binding of other biomolecules to the fibril surfaces. The reduced fibrillation rates observed for Aβ in CSF [44] is indeed in line with that assumption. Finally, it is also important to move on and explore how other biomolecules, e.g. lipids or proteoglycans, are incorporated in the plaques and to investigate the sequestration of intracellular components and whole cells, as illustrated in Fig. 5.

Embracing the multiprotein nature of amyloid deposits also expand the therapeutic opportunities. All proteins on the list are potential drug targets. Therapeutic effects could be achieved by inhibit binding or by modulating protein functions to restore loss-of-function effects or counteract toxic gain-of-function effects. Having the highly connected functional network in mind, even small alterations in the composition of plaques could have significant effects on the pathology.

## Data Availability

Not applicable

## List of abbreviations

α2M: Alpha-2-macroglobulin
Aβ: Amyloid β
A1AT: Alpha-1-antitrypsin
ACT: Alpha 1-antichymotrypsin
AD: Alzheimer’s disease
APLP1: Amyloid-like protein 1
ApoA-I: Apolipoprotein A-I
ApoA-II: Apolipoprotein A-II
ApoA-VI: Apolipoprotein A-VI
ApoB-100: Apolipoprotein B-100
ApoC-I: Apolipoprotein C-I
ApoC-II: Apolipoprotein C-II
ApoC-III: Apolipoprotein C-III
ApoC-IV: Apolipoprotein C-IV
ApoD: Apolipoprotein D
ApoE: Apolipoprotein E
ApoJ: Apolipoprotein J
APP: Amyloid β precursor protein
C1q: Complement 1q
C1r: Complement 1r
C1s: Complement 1s
C3: Complement 3
C3aR1: Complement 3a receptor-1
C4: Complement 4
C5: Complement 5
C7: Complement 7
CAA: Cerebral amyloid angiopathy
CD: Circular dichroism
CLU: Clusterin
CNS: Central nervous system
CRTAC1: Cartilage acidic protein 1
CSF: Cerebrospinal fluid
CysC: Cystatin C
ECM: Extracellular matrix
ELISA: Enzyme linked immunosorbent assay
ENO1: Alpha-enolase
G3BP: Galectin-3-binding protein
GAPDH: Glyceraldehyde-3-phosphate dehydrogenase
Gpc-1: Glypican-1
Hb: Hemoglobin
HDL: High-density lipoproteins
Hpt: Haptoglobin
Hpx: Hemopexin
IAPP: Islet amyloid polypeptide
ITIH2: Inter-alpha-trypsin inhibitor heavy chain H2
LC-MS/MS: Liquid chromatography coupled to tandem mass spectrometry
LCM: Laser capture microdissection
LRP2: Lipoprotein-related protein 2
MRM: Multiple reaction monitoring
NAC: Non amyloid-β component
NCAM1: Neural cell adhesion molecule 1
NMR: Nuclear magnetic resonance
OPN: Osteopontin
PCPE 1: Procollagen C-endopeptidase enhancer 1
PET: Positron emission tomography
PGK1: Phosphoglycerate kinase 1
SAP: Serum amyloid P component
SEC: Size exclusion chromatography
SPR: Surface plasmon resonance
TEM: Transmission electron microscopy
TREM2: Triggering receptor expressed on myeloid cells 2
TTR: Transthyretin
WB: Western blot
